# Internal Tooth Structure and Burial Practices: Insights into the Neolithic Necropolis of Gurgy (France, 5100-4000 cal. BC)

**DOI:** 10.1371/journal.pone.0159688

**Published:** 2016-07-22

**Authors:** Mona Le Luyer, Michael Coquerelle, Stéphane Rottier, Priscilla Bayle

**Affiliations:** 1 Unité Mixte de Recherche 5199, de la Préhistoire à l’Actuel: Culture, Environnement, Anthropologie (UMR 5199 PACEA), Université de Bordeaux, Pessac, France; 2 Department of Oral Surgery, Rey Juan Carlos University, Alcorcon, Spain; Université de Poitiers, FRANCE

## Abstract

Variations in the dental crown form are widely studied to interpret evolutionary changes in primates as well as to assess affinities among human archeological populations. Compared to external metrics of dental crown size and shape, variables including the internal structures such as enamel thickness, tissue proportions, and the three-dimensional shape of enamel-dentin junction (EDJ), have been described as powerful measurements to study taxonomy, phylogenetic relationships, dietary, and/or developmental patterns. In addition to providing good estimate of phenotypic distances within/across archeological samples, these internal tooth variables may help to understand phylogenetic, functional, and developmental underlying causes of variation. In this study, a high resolution microtomographic-based record of upper permanent second molars from 20 Neolithic individuals of the necropolis of Gurgy (France) was applied to evaluate the intrasite phenotypic variation in crown tissue proportions, thickness and distribution of enamel, and EDJ shape. The study aims to compare interindividual dental variations with burial practices and chronocultural parameters, and suggest underlying causes of these dental variations. From the non-invasive characterization of internal tooth structure, differences have been found between individuals buried in pits with alcove and those buried in pits with container and pits with wattling. Additionally, individuals from early and recent phases of the necropolis have been distinguished from those of the principal phase from their crown tissue proportions and EDJ shape. The results suggest that the internal tooth structure may be a reliable proxy to track groups sharing similar chronocultural and burial practices. In particular, from the EDJ shape analysis, individuals buried in an alcove shared a reduction of the distolingual dentin horn tip (corresponding to the hypocone). Environmental, developmental and/or functional underlying causes might be suggested for the origin of phenotypic differences shared by these individuals buried in alcoves.

## Introduction

Traditionally, biological affinities and kinship between populations and individuals are approached by the analysis of crown size and shape, using external diameters and non-metric variations [1–15]. Crown size and shape are influenced by genetic, epigenetic and environmental factors (see [16,17] for an overview). Whereas the polygenetic control is relatively strong [5,17–21] and seems to be dominant for crown size [18], the impact of environmental factors on tooth variations has been demonstrated, notably by the studies of twins [19,22–25]. In addition, the buccolingual diameter is less influenced by environmental factors than the mesiodistal diameter [26], suggesting a different genetic control between these two dimensions [27,28]. Non-metric variations are also determined by multiple factors, which are under moderate to high-genetic and environmental controls [7,29–31]. They are considered as reliable markers for measuring biological relatedness and thus widely used to estimate biological relationships between populations and affinities between individuals [9,11,31–46].

Community-shared or family-centered practices might influence the organization of the necropolises, this means that burial practices may reflect the social composition of the population [31,33,47]. Morphological and genetic methods are generally applied in order to characterize biological relationships between individuals in burial grounds. It is possible to identify closely related individuals but the precise genealogical degree of this kinship is rarely specified.

Because of the high heritability of non-metric variations [16,31], these phenotypic data are studied in order to assess the social structure of necropolises [31,44,47–49]. In particular, teeth are often singled out for their potential to identify biological relatives [50–52] and even siblings [15]. More recently, advances in paleogenetics allow to compare cemetery organization and kinship from ancient DNA analysis, also with some precise genetic affiliation [53–56]. Affinities obtained from these two methods–non-metric and genetic–are correlated [57–59]. Paleogenetic analyses are destructive and dependent on the preservation of the remains–ancient DNA may not be conserved. So far, external morphometric analyses of the crown are used to assess biological affinities, providing information that describes in essence the macrostructural outcomes of dental development.

At meso- and microstructural level, teeth record a wealth of unique information within their tissues that are crucial in bioarcheology and paleoanthropology [60–80]. Enamel thickness, crown tissue proportions and enamel-dentin junction (EDJ) shape have been shown as relevant parameters to finely characterize taxonomy, phylogenetic relationships, dietary and/or developmental patterns [62–69,81–95]. The EDJ, which is the interface between the enamel cap and crown dentin [96] and the developmental precursor and primary contributor to the outer morphology of the crown [65,91,92,97,98], has been shown to successfully discriminate hominoid species [62,65,91,92,99–102]. Moreover, the EDJ is morphologically more conservative than the outer enamel surface and provides essential information about the developmental processes underlying tooth crown growth [65,100,102]. According to the inhibitory cascade model of development [103], the morphology of the crown is an iterative process determined by a morphodynamic interaction between developmental genes and cusps morphogenesis. This model predicts the future cusp size and shape, the number of cusps [104–107] as well as the pattern of tooth sizes for the lower postcanine teeth [108].

Advanced virtual imaging techniques, that are non-invasive methods, allow such quantitative and qualitative characterizations of internal structures. However, at a microevolutionary scale, these variables have been poorly assessed in modern humans to discuss inter- or intrapopulational variability [109,110]. While the study of internal tooth structure may help to assess subtle phenotypic differences across/within archeological assemblages, in our knowledge, the use of internal tooth structure is original to characterize the variations at the intrasite scale. More importantly, in addition to provide precise phenotypic assessment, internal tooth structure analyses may allow to understand the developmental, functional and phylogenetic underlying causes of these variations.

### The Neolithic necropolis of Gurgy

**Fig 1:**
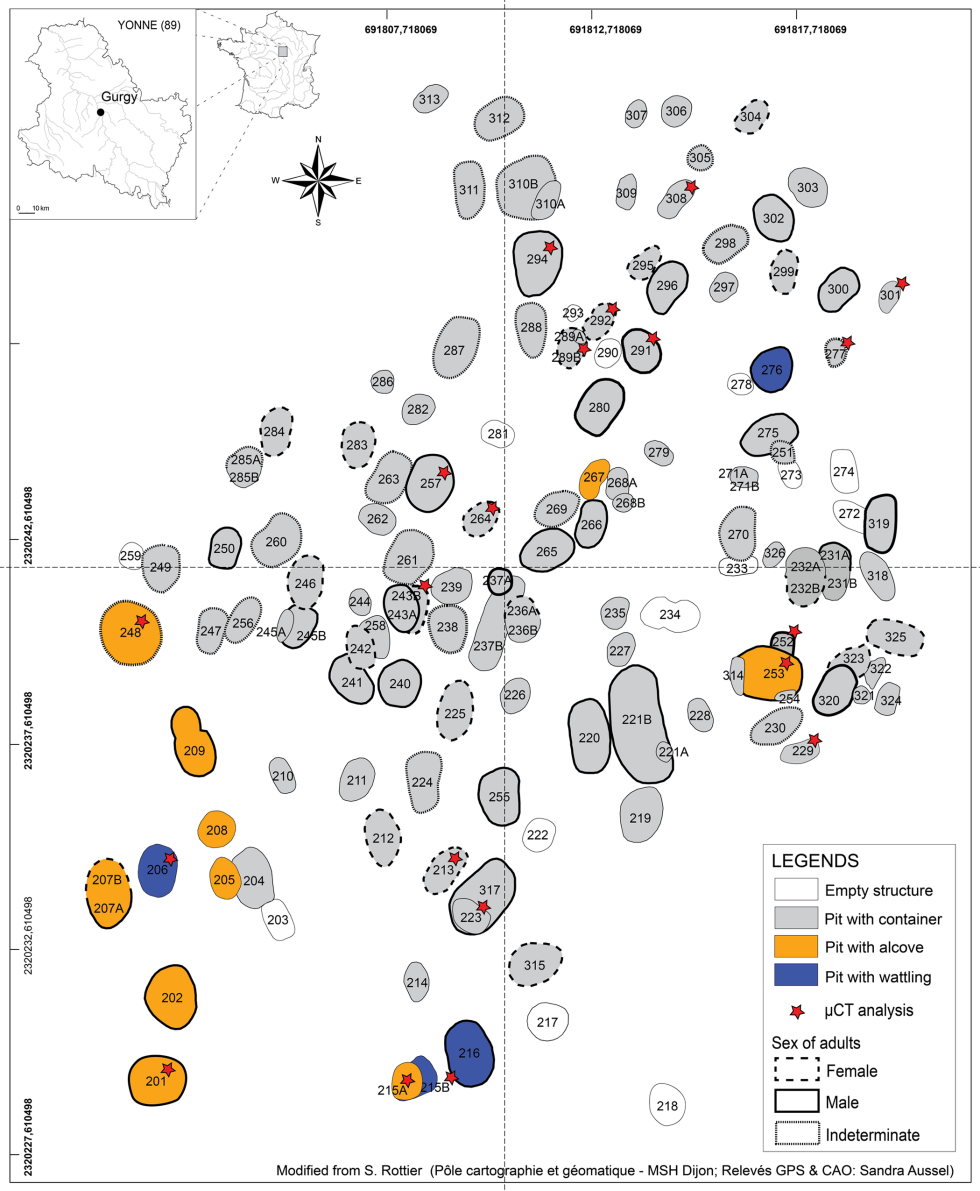
Map of the Neolithic necropolis of Gurgy (Yonne, France).

Discovered in 1997 in the southern Paris Basin (Yonne, France), excavations at Gurgy yielded 128 individuals (**Fig 1**), which make the site one of the most important necropolises currently known for the Early/Middle Neolithic transition in Western Europe [111,112]. Among the 134 pits dug in Gurgy, 120 were identified as primary burials: individual structures were largely dominant and few were double burials. Both individual and double burials present various funerary architectures [112], including pits with evidence of a container in perishable material (container), pits with a wattle-like construction on the walls (wattling) and pits with a lateral overdigging where the body was deposited (alcove, see **S1 Fig**). The majority of the bodies were positioned on the left side, with the head to the south or the south-west, upper limbs hyperflexed and lower limbs either bended or hyperbended [113]. Some grave goods were found associated with the individuals buried at Gurgy: animal bones or teeth, shells, flint, ochre and rare pottery [111]. Radiocarbon dates of human remains range between 5100–4000 cal. BC (see [114]) and represent a continuous use of the necropolis for a thousand years. Three main chronological phases of occupation of the site could be identified: the principal phase (B) with the most intensive use is ranged from 4800 and 4500 cal. BC, is framed by an early phase (A) before 4800 cal. BC and a recent phase (C) dated after 4500 cal. BC (see **S2 Fig**).

The necropolis of Gurgy is situated at the confluence of the two migration routes of the European Neolithization, in the culturally rich and complex archeological area of the Paris Basin during the transition from the Ancient to the Middle Neolithic. Given these multiple cultural influences are visible at Gurgy [111,112], it is not possible to propose a specific cultural attribution ([113,114] see in particular the Supplementary Informations). Indeed, for the period and the region, Gurgy revealed a more homogeneous and inconspicuous funerary profile: a necropolis without monument and any structuring of funerary space [44,115]. Paleogenetic analyses show equivalent genetic contributions of the two European neolithization waves in Gurgy population, revealing the most ancient mixture between farmers from both Danubian and Mediterranean migration routes [114]. At the intrapopulation scale, even if ancient DNA was not conserved in all sampled individuals and no Y chromosome DNA was found, and thus no precise degrees of kinship were discussed, some individuals sharing mitochondrial DNA haplotypes and archeological and spatial features might be closely maternally related [114,116].

According to the analyses conducted so far, the Gurgy population is homogeneous regarding isotopic variation [117], enamel thickness topography and tooth wear patterns [93]. Although influenced by the rich cultural diversity of the Paris Basin [113,114], the funerary practices at Gurgy are rather homogeneous [113]. All these data suggest that the necropolis was used by a uniform population (even if genetically derived from both Neolithization waves) sharing a common cultural framework.

### Aims of the study

In this study, a high resolution microtomographic (microCT)-based record of upper permanent second molars from Neolithic individuals of the necropolis of Gurgy was applied with the aim to evaluate intrasite variation in crown tissue proportions, thickness and distribution of enamel, and enamel-dentin junction shape. This study explore subtle phenotypic dental variation and its underlying causes among individuals buried at Gurgy. Using these internal tooth structure parameters and facing these data against burial practices and chronocultural parameters, this investigation will address the following questions:

- Do phenotypic distances obtained from the study of morphological and metrical variation of internal tooth structure are a reliable proxy to track groups sharing similar chronocultural and burial practices?
- Is one of these internal parameters more relevant to discuss interindividual variability?
- Can causes of dental variations be suggested?

## Materials and Methods

### Samples

For this preliminary study, 20 upper permanent second molars (UM2) were examined for 13 adults (whom 6 males, 5 females, and 2 indeterminate) and 7 immature individuals from Gurgy (**Table 1**), all buried in primary structures (**Fig 1**). UM2 have been selected because they are often less worn than M1, and their development is more correlated to somatic and sexual maturation than those of M3 [118]. Crowns are complete (at least, maturational stage D and further [119]) and well-preserved, exempt from caries, pathologies and damages. Dental wear patterns were recorded according to the procedure of Molnar [120] and teeth range from unworn to slightly worn (**Table 1**). Age-at-death estimation was based on dental development [121,122], bone ossification and diaphyseal length [123] and chronological metamorphosis of the auricular surface of the ilium [124]. Sex was assessed using the morphology and morphometry of the *ossa coxae* [125–127]. Details on sample composition regarding biological and chronocultural parameters are given in **Table 1,** and summarized on the necropolis map (**Fig 1**). Direct radiocarbon ages of human remains were available for a large sample of individuals in the necropolis (calibrated at 2 sigmas with OxCal 4.2.4 [128] and curve IntCal13 [129], see also **S2 Fig**)

**Table 1:** The 20 Neolithic individuals sampled in the necropolis of Gurgy. Details of biological (age and sex, maturational and wear stages, mtDNA haplogroups) and chronocultural parameters (radiocarbon age, phase, burial structure, orientation and location in the necropolis).

Individual ^1^			Teeth			mtDNA	Burial			Ages	
Number	Age ^2^	Sex ^2^	UM2	Maturational stage ^3^	Wear stage ^4^	Haplogroug ^5^	Structure ^6^	Orientation	Location	14C cal. BC	Phase ^7^
**201**	16–18	M	left	H	2	K	Alcove	E_W	SW	5206–4840	A
**206**	3.5–7		left	D	1		Wattling	N_S	SW		
**213**	15–29	F	right	H	2		Container	NE_SW	SW	4937–4728	B
**215A**	4–8		right	D	1		Alcove	NW_SE	SW		B
**215B**	8–14		right	E	1	J1	Wattling	N_S	SW		
**223**	4–7		right	D	1	U5	Container	N_S	SW	4770–4536	B
**229**	12–19		right	H	1	K	Container	SW_NE	SE		C
**243B**	>20	F	left	H	3	X	Container	N_S	SW	4828–4609	B
**248**	>40	I	left	H	3	U5	Alcove	N_S	SW	4313–3991	C
**252**	>30	M	left	H	1		Container	N_S	SE	4763–4536	B
**253**	20–59	M	left	H	2		Alcove	NE_SW	SE	4235–3991	C
**257**	>30	M	right	H	3		Container	NE_SW	NW	4841–4556	B
**264**	15–29	F	left	H	2	H1	Container	NE_SW	NW		
**277**	15–29	I	right	H	2	J	Container	N_S	NE		
**289B**	>30	F	right	H	1	K	Container	SW_NE	NE	4770–4489	B
**291**	>30	M	right	H	3		Container	NE_SW	NE		B
**292**	20–29	F	right	H	1	H1	Container	SW_NE	NE		B
**294**	>20	M	left	H	2	U5	Container	E_W	NE		B
**301**	6–12		left	E	1	K	Container	NE_SW	NE	4763–4536	B
**308**	9–14		right	G	1		Container	NE_SW	NE		

^1^ The original specimens are deposited in the Ostéothèque de Pessac (Université de Bordeaux). All material included is archeological; no permits were required for the described study.

^2^ Revised from [113,114]; personal communications and observations; M = male; F = female; I = indeterminate.

^3^ According to Demirjian *et al*. [119], D = the crown formation is completed down to the enamel-dentin junction; E = the root length is still less than the crown height; F = the root length is equal to or greater than the crown height; G = the walls of the root canal are parallel and its apical end is still partially open; H = the apical end of the root canal is completely closed.

^4^ According to Molnar [120], 1 = unworn teeth; 2 = minimal wear facets; 3 = small dentin patches.

^5^ All individuals were selected for mitochondrial DNA (mtDNA) analysis, unfortunately, not all samples yielded results. See Rivollat *et al*. [114].

^6^ Burial pictures are provided in **S1 Fig**. See Rottier [112] for description.

^7^ A: early phase (before 4800 cal. BC); B: principal phase (4800–4500 cal. BC); C: recent phase (after 4500 cal. BC). See also **S2 Fig**.

### Microtomographic record

The UM2 were scanned on Skyscan 1076 X-ray equipment set at the MRI platform (University Montpellier 2, France). Acquisitions were realized according to the following parameters: 100 kV voltage, 100 μA current, a 1.0 mm aluminum filter and a rotation step each 0.20°. The software Nrecon v1.6.6 (Skyscan) was used to reconstruct the final volumes with an isotropic voxel size ranging from 17.93 μm for isolated teeth to 36.18 μm for jaw fragments. Following the half-maximum height method [130,131], a semi-automatic threshold-based segmentation with manual corrections was conducted using Avizo v.7 (VSG) [62,84–87,89,94,132–135]. Crowns were digitally isolated from roots [85] and three-dimensional (3D) surface models were generated using a constrained smoothing algorithm. All 3D surface models were deposited in MorphoMuseuM [136].

### Dental tissue proportions and enamel thickness analysis

Using MPSAK v2.9 (developed by L. Bondioli, available in [137], seven 2D variables were measured or calculated on virtual buccolingual cross-sections realized through the dentin horn tips of the mesial cusps (see **S3 Fig**): total crown area (a, mm^2^); enamel area (c, mm^2^); coronal dentin and pulp area (b, mm^2^); percentage of crown area that is dentin and pulp (%b, %); enamel-dentin junction (EDJ) length (e, mm); 2D average enamel thickness (AET2D (= c/e), mm) and 2D relative enamel thickness (RET2D (= AET2Dx100/(b)^1/2^), scale-free) [81,86]. For lightly worn crowns, 2D corrections of outer enamel surface were made prior to measurements of the AET2D and RET2D values: reconstructions of the removed enamel were performed based on morphology observed for unworn teeth [69].

Height variables describing 3D dental tissue proportions were digitally measured or extracted using Avizo v.7 (VSG) on the crown reconstructions (see **S4 Fig**): total crown volume (Cvol, mm^3^); enamel volume (Evol, mm^3^); coronal dentin volume (Dvol, mm^3^); coronal dentin and pulp volume (DPvol, mm^3^); percentage of crown volume that is dentin and pulp (%DPvol, %); EDJ surface area (S_EDJ_, mm^2^); 3D average enamel thickness (AET3D (= Evol/S_EDJ_), mm) and 3D relative enamel thickness (RET3D (= AET3Dx100/(DPvol)^1/3^), scale-free) [62,83–85]. While enamel thickness increases allometrically with body and/or tooth size, RET3D allows comparisons between specimens with different body size [81].

The topographic variation of standardized enamel thickness (see **S3 Fig**) was measured using MPSAK v2.9 [137] in buccal and lingual aspects of the virtual buccolingual cross-sections [93,138]. In order to calibrate enamel thickness values and allow comparisons independent to tooth size and occlusal wear, the bi-cervical diameter (BCD) was defined at 10 mm (**S3 Fig**) prior to digitize thicknesses between enamel-dentin junction and outer enamel surface, from the neck to the apex with an interval of 0.25 mm [93,138].

Three-dimensional maps of the topographic distribution of enamel thickness were created by measuring the distance between the outer enamel surface and enamel-dentin junction [64]. Differences in enamel thickness were rendered by a thickness-related, pseudo-color scale ranging from dark blue for thinner enamel to red for thicker enamel.

Estimated intra- and interobserver error rates were lower than 5% in all measured variables. The non-parametric Mann–Whitney U-test was employed to evaluate differences between the sexes and between individuals sharing cultural parameters. Plots of AET against b or DPvol, respectively in 2D and in 3D, were used to illustrate the relationship between AET and tooth size. Cluster analyses were performed on 2D and 3D tissue proportions using Ward’s hierarchical clustering method [139], with a bootstrap of 1000 repetitions.

### Geometric morphometric analysis of EDJ shape

Using the software Viewbox 4 (dHAL software, Kifissia, Greece), a 3D template of 114 points was created with an assemblage of three sets of 3D landmarks (**S5 Fig**). Compared to studies aiming to assess variations at a macroevolutionary scale [91,95], a large number of landmarks and semilandmarks was placed in order to finely quantify morphological EDJ variations in a microevolutionary context. The first set includes five anatomical landmark points: four were digitized on the tip of the dentin horn of corresponding four main cusps (i.e. protocone, paracone, metacone, hypocone) and one on the maximum lower part of the occlusal basin. Although unworn to slightly worn UM2 were selected, four teeth exhibit small dentin patches. Based on morphology observed for preserved dentin horns, reconstructions of the apex of dentin horn tips were made using Avizo v.7 (VSG), prior to digitally place landmarks. The second set is composed of 52 curve semilandmarks: 17 were digitized along the top of the ridges which connected the three dentin horns of the protocone, the paracone and the metacone, five were digitized along the ridge between the metacone and the hypocone, and 30 were collected along the cervix of the tooth crown. The third set includes 57 surface semilandmarks distributed uniformly over the occlusal basin and the wall of the EDJ surface [91,101,140–143].

Using Viewbox 4 (dHAL), the template was warped onto each specimen’s EDJ surface by a thin-plate spline (TPS) interpolation function, and points were projected onto the targeted EDJ surface. As part of the digitization process, semilandmarks were allowed to slide along the curves and surfaces in order to minimize the bending energy of the TPS computed between each EDJ specimen and the Procrustes average shape [140,144]. After sliding, landmarks and semilandmarks were treated as homologous points [140] and converted to shape coordinates by Generalized Procrustes Analysis [144–146]. This involves rescaling the landmark coordinates so that each configuration has a unit Centroid Size (CS). Next, all configurations were translated and rotated to minimize the overall sum of the squared distances between corresponding (semi)landmarks.

Using R ([147], packages Morpho, shapes, scatterplot3d), a Principal Component Analysis (PCA) was carried out on the matrix of shape coordinates augmented by a column of the natural logarithm of Centroid Size (LnCS)–corresponding to a PCA in form space [148]. PC1 usually captures overall size variation as well as size-related shape variation (allometry), whereas the other PCs contain residual, non-allometric, shape variation and are weakly correlated with size.

A 3D digital EDJ surface was warped towards the Procrustes mean form using a thin plate spline (TPS) interpolation function using Avizo v.7 (VSG). Thereafter, the surface of the Procrustes mean configuration (consensus) was used to visualize size and shape variation along the PCs. The shape deformation represented by the eigenvectors of a particular PC was visualized as a TPS deformation from the consensus plus or minus the eigenvectors (right and left sides of the PC, respectively). Once the eigenvectors (those related to the shape variables) are added or subtracted from the consensus, all variables are also multiplied by the exponent of the eigenvector for LnCS [149].

## Results

### Dental tissue proportions and enamel thickness

**Fig 2:**
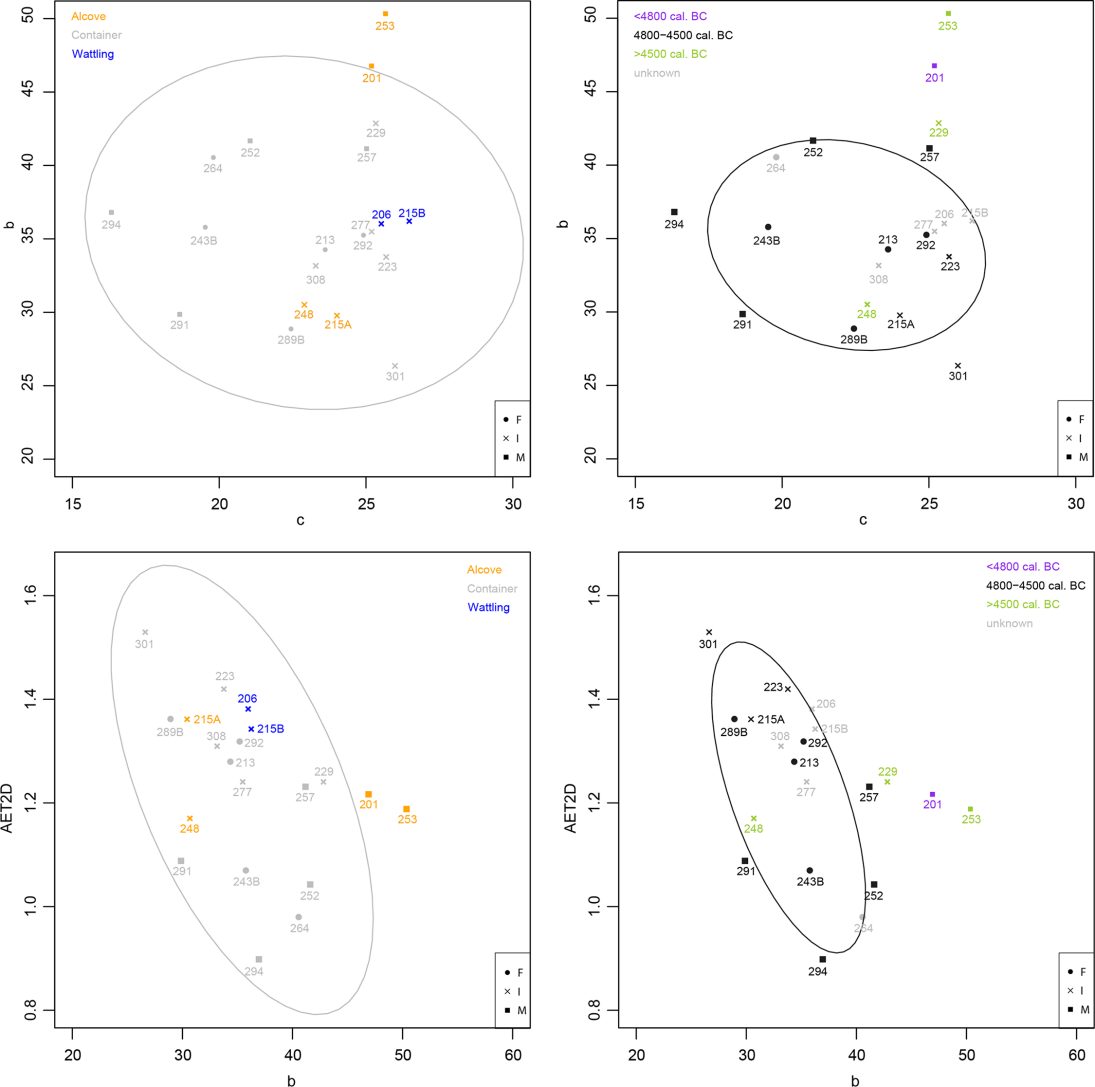
Plot of c against b (top) and plot of b against AET2D (graphic representation of RET2D, bottom) according to burial (left) and phase (right).

For each individual, 2D variables measured on virtual buccolingual sections–and corrected for occlusal wear–are presented in **Table 2**. While males tend to have higher 2D tissue proportions, in particular higher dentin surface (**Table 3,** see also **S6 Fig**), no significant differences were found between sexes, burial structures and occupation phases of the necropolis. In majority, the same information is found with 3D and 2D crown variables, but plots of 2D tissue proportions show slightly more nuanced differences between burials and between phases (**Fig 2)**. Indeed, only the males buried in a pit with alcove (individuals 201 and 253) fall out of the variability shown by individuals buried in a pit with container (**Fig 2 left**), while individual 248 which is from the recent phase appears to be included in the variability of individuals from the main phase (**Fig 2 right**).

**Table 2:** Measured 2D variables of crown tissue proportions for each individual.

Individual	a	c	b	%b	e	AET2D	RET2D
**201**	72.19	25.21	46.98	65.08	20.66	1.22	17.80
**206**	61.58	25.53	36.05	58.54	18.53	1.38	22.95
**213**	57.90	23.61	34.29	59.22	18.40	1.28	21.91
**215A**	54.16	24.06	30.10	55.58	17.72	1.36	24.75
**215B**	62.53	26.37	36.16	57.83	19.75	1.34	22.20
**223**	59.41	25.68	33.73	56.77	18.09	1.42	24.45
**229**	68.17	25.34	42.83	62.83	20.51	1.24	18.88
**243B**	55.32	19.52	35.80	64.71	18.20	1.07	17.93
**248**	53.67	22.99	30.68	57.16	19.65	1.17	21.12
**252**	62.77	21.06	41.71	66.45	20.34	1.04	16.04
**253**	75.97	25.65	50.32	66.24	21.64	1.19	16.71
**257**	66.19	25.03	41.16	62.18	20.35	1.23	19.17
**264**	60.31	19.79	40.52	67.19	20.18	0.98	15.41
**277**	60.64	25.19	35.45	58.46	20.28	1.24	20.86
**289B**	51.30	22.44	28.86	56.26	16.45	1.36	25.39
**291**	48.52	18.66	29.86	61.54	17.04	1.09	20.04
**292**	60.15	24.92	35.23	58.57	18.94	1.32	22.17
**294**	53.11	16.33	36.79	69.26	18.11	0.90	14.87
**301**	52.58	26.01	26.57	50.53	17.01	1.53	29.66
**308**	56.44	23.29	33.15	58.73	17.78	1.31	22.75

a = total crown area (mm^2^)

c = enamel area (mm^2^)

b = coronal dentin and pulp area (mm^2^)

%b = percentage of crown area that is dentin and pulp (%)

e = enamel-dentin junction length (mm)

AET2D = 2D average enamel thickness (mm)

RET2D = 2D relative enamel thickness (scale free).

**Table 3:** Descriptive statistics of 2D measured variables for all individuals, female and male individuals, separately.

		a	c	b	%b	e	AET2D	RET2D
**All individuals**	mean ± SD	59.64 ± 7.06	23.33 ± 2.84	36.31 ± 6.11	60.66 ± 4.74	18.98 ± 1.45	1.23 ± 0.16	20.75 ± 3.77
	range	48.52–75.97	13.66–26.37	26.57–50.32	50.53–69.26	16.45–21.64	0.90–1.53	14.87–29.66
	CV	0.12	0.12	0.17	0.08	0.08	0.13	0.18
**Female**	mean ± SD	57.00 ± 3.77	22.06 ± 2.36	34.94 ± 4.16	61.19 ± 4.57	18.43 ± 1.35	1.20 ± 0.17	20.56 ± 3.91
	range	51.30–60.31	19.52–24.92	28.86–40.52	56.26–67.19	16.45–20.18	0.98–1.36	15.41–25.39
	CV	0.07	0.11	0.12	0.07	0.07	0.14	0.19
**Male**	mean ± SD	63.16 ± 10.68	21.99 ± 3.92	41.14 ± 7.28	65.12 ± 2.88	19.69 ± 1.74	1.11 ± 0.13	17.44 ± 1.95
	range	48.52–75.97	16.33–25.65	29.86–50.32	61.54–69.26	17.04–21.64	0.90–1.23	14.87–20.04
	CV	0.17	0.18	0.18	0.04	0.09	0.12	0.11

For the definitions of the abbreviated variables, see **Table 2.**

All 3D variables measured for upper permanent second molars (UM2) of each specimen are presented in **Table 4**and descriptive statistics are detailed in **Table 5**. Reconstructions of 3D surface models for enamel and dentin components are shown **S7 Fig**, which allows to compare the morphology between the UM2 crowns.

**Table 4:** Measured 3D variables of crown tissue proportions for each individual.

Individual	Cvol	Evol	Dvol	DPvol	%DPvol	S_EDJ_	AET3D	RET3D
**201**	565.30	268.09	281.42	297.21	52.58	201.57	1.33	19.93
**206**	410.86	209.73	168.26	201.13	48.95	159.34	1.32	22.47
**213**	383.43	199.18	182.83	184.25	48.05	150.36	1.32	23.28
**215A**	330.23	180.57	141.20	149.66	45.32	132.39	1.36	25.69
**215B**	401.50	208.45	179.16	193.05	48.08	158.93	1.31	22.69
**223**	442.63	234.91	141.24	207.72	46.93	161.83	1.45	24.51
**229**	478.58	240.55	237.69	238.03	49.74	184.39	1.30	21.05
**243B**	363.94	144.34	214.76	219.60	60.34	176.23	0.82	13.58
**248**	279.20	136.33	140.97	142.87	51.17	131.06	1.04	19.90
**252**	399.21	193.60	203.33	205.62	51.51	157.67	1.23	20.80
**253**	474.74	238.93	230.92	235.81	49.67	175.41	1.36	22.05
**257**	429.40	210.53	216.30	218.88	50.97	175.29	1.20	19.93
**264**	380.95	187.12	189.12	193.83	50.88	158.48	1.18	20.40
**277**	424.31	229.00	189.61	195.31	46.03	169.25	1.35	23.32
**289B**	370.49	214.24	150.01	156.25	42.17	138.42	1.55	28.74
**291**	314.69	127.33	186.40	187.35	59.54	145.39	0.88	15.31
**292**	428.73	230.34	196.17	198.39	46.27	162.34	1.42	24.33
**294**	383.65	149.97	228.08	233.68	60.91	176.03	0.85	13.83
**301**	424.02	250.02	171.59	174.00	41.04	153.83	1.63	29.11
**308**	375.34	212.61	161.17	162.73	43.36	140.57	1.51	27.70

Cvol = total crown volume (mm^3^)

Evol = enamel volume (mm^3^); DPvol = coronal dentin and pulp volume (mm^3^)

%DPvol = percentage of crown that is dentin and pulp (%)

S_EDJ_ = enamel-dentin junction area (mm^2^)

AET3D = 3D average enamel thickness (mm)

RET3D = 3D relative enamel thickness (scale free).

**Table 5:** Descriptive statistics of measured 3D variables for all individuals, female and male individuals, separately.

		Cvol	Evol	Dvol	DPvol	%DPvol	S_EDJ_	AET3D	RET3D
**All individuals**	mean ± SD	403.06 ± 62.46	203.29 ± 39.22	190.51 ± 36.86	199.77 ± 35.87	49.83 ± 5.50	160.44 ± 18.07	1.27 ± 0.22	21.93 ± 4.34
	range	279.20–565.30	127.33–268.09	140.97–281.42	142.87–297.21	41.04–60.91	131.06–201.57	0.82–1.63	13.58–29.11
	CV	0.15	0.19	0.19	0.18	0.11	0.11	0.18	0.20
**Female**	mean ± SD	385.51 ± 25.42	195.04 ± 32.65	186.58 ± 23.69	190.46 ± 23.09	49.54 ± 6.81	157.17 ± 14.05	1.26 ± 0.28	22.06 ± 5.61
	range	363.94–428.73	144.34–230.34	150.01–214.76	156.25–219.60	42.17–60.34	138.42–176.23	0.82–1.55	13.58–28.74
	CV	0.07	0.17	0.13	0.12	0.14	0.09	0.22	0.25
**Male**	mean ± SD	427.83 ± 85.64	198.07 ± 53.04	224.41 ± 32.44	229.76 ± 37.69	54.19 ± 4.78	171.89 ± 19.11	1.14 ± 0.22	18.64 ± 3.28
	range	314.69–565.30	127.33–268.09	186.40–281.42	187.35–297.21	49.67–60.91	145.39–201.57	0.85–1.36	13.83–22.05
	CV	0.20	0.27	0.14	0.16	0.09	0.11	0.20	0.18

For the definitions of the abbreviated variables, see **Table 4.**

**Fig 3:**
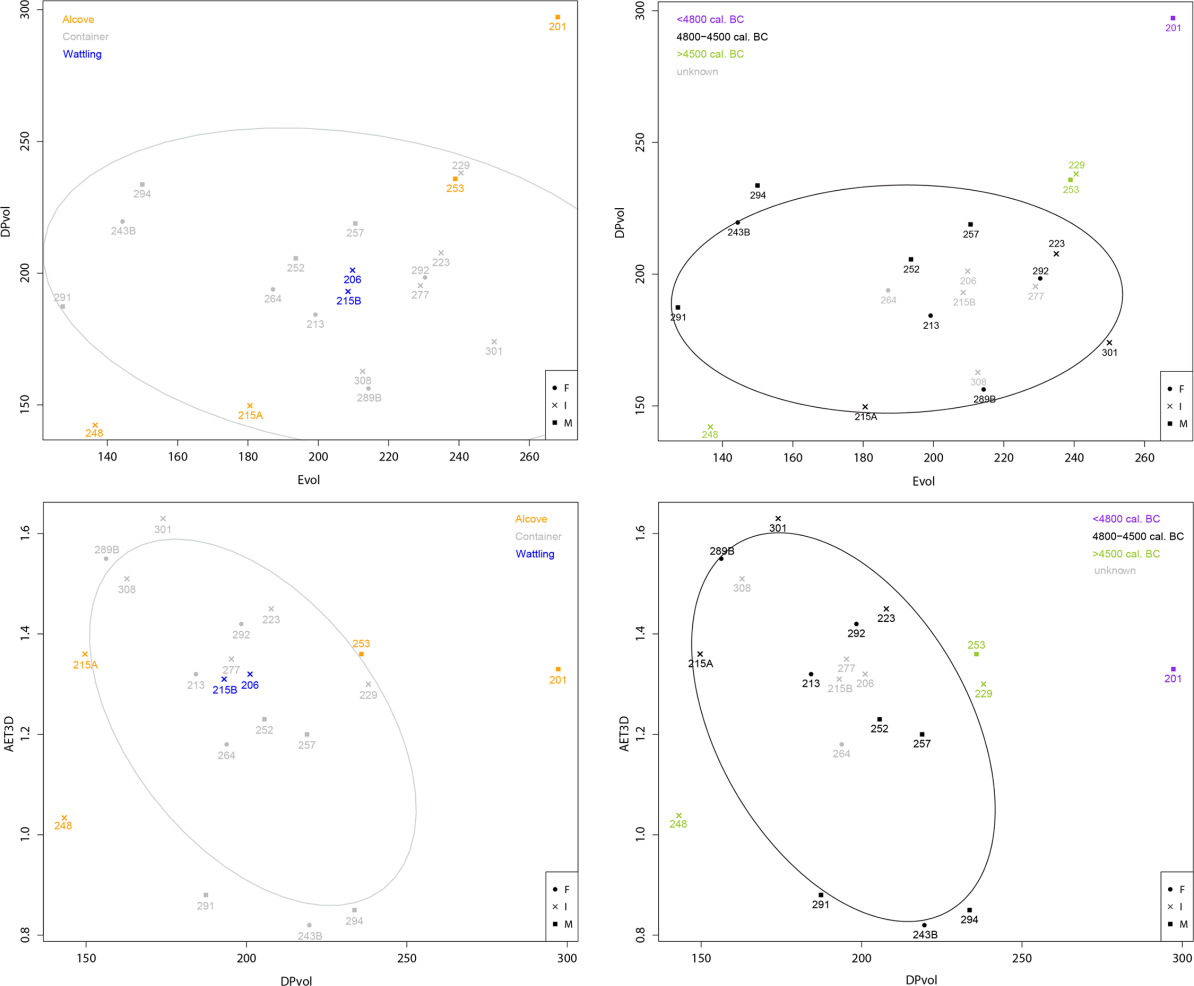
Plot of Evol against DPvol (top) and plot of DPvol against AET3D (graphic representation of RET3D, bottom) according to burial (left) and phase (right).

The results show an overall biological proximity in enamel thickness and dental tissue proportions for individuals buried at Gurgy. Males show greater quantity of each tissue than females (**Table 5**). From Mann-Whitney U-test, only the volume of dentin (Dvol) is significantly higher for males than for females (p = 0.045). Proportion of these tissues into the crown (%DPvol) is comparable between sexes. No significant differences were found between individuals buried in different burials structure or individuals from different phases of the necropolis. However, while relatively low range of variation is shown in dental tissue proportions and enamel thickness for individuals of Gurgy (**Fig 3**), males tend to have higher crown tissue proportions than females (see also **S8 Fig**). Also, even if occlusal wear slightly affects enamel volume (Evol), grouping of individuals based on tissue proportions and thickness show an overall correlation with chronocultural parameters (**Fig 3**). Individuals buried in pit with wattling are systematically included in the variability shown by those buried in pit with container, whereas individuals buried in alcove are out of this variability, in particular individual 201 and 248. Moreover, individuals buried in a pit with wattling (206-215B) are very close and share high proximity in crown tissue proportions (Evol and DPvol, **Fig 3 top left**) and enamel thickness (**Fig 3 bottom left**), while those buried in pit with alcove present the highest variability (individuals 248-215A and 201 are at extreme opposition, respectively, **Fig 3 left**). Also, individuals from the early phase (A) and the recent phase (C) of the necropolis use are out of the biological variability observed for individuals from the principal occupation of the necropolis (phase B, **Fig 3 right**). Particularly, individual 201 from the oldest phase exhibits the most different condition, as he presents the highest value for both Evol and DPvol (**Fig 3 top right**) and EDJ surface (**Fig 3 bottom right**).

**Fig 4:**
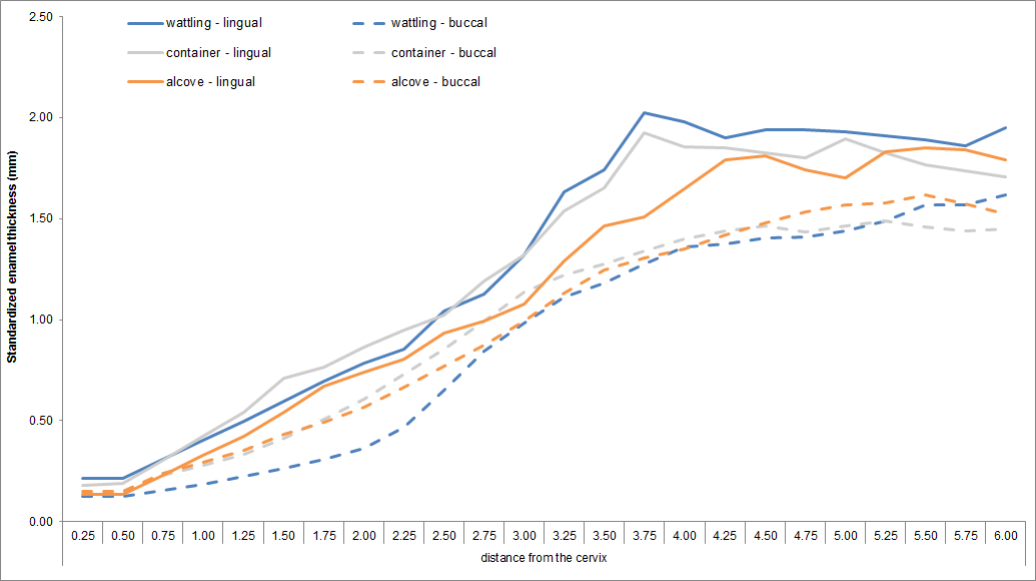
Topographic variation of standardized enamel thickness measured on the lingual (continuous lines) and buccal (dotted lines) aspects according to burial structures.

Measurements of topographic variation in enamel thickness allow comparisons which are not affected by occlusal wear. For all individuals, enamel is thicker on the lingual aspect than on the buccal aspect (**Fig 4**). Individuals buried in a pit with alcove show a more homogeneous enamel thickness distribution between lingual and buccal aspects of the crown, and present systematically thinner enamel on the lingual aspect than other individuals (**Fig 4**).

**Fig 5:**
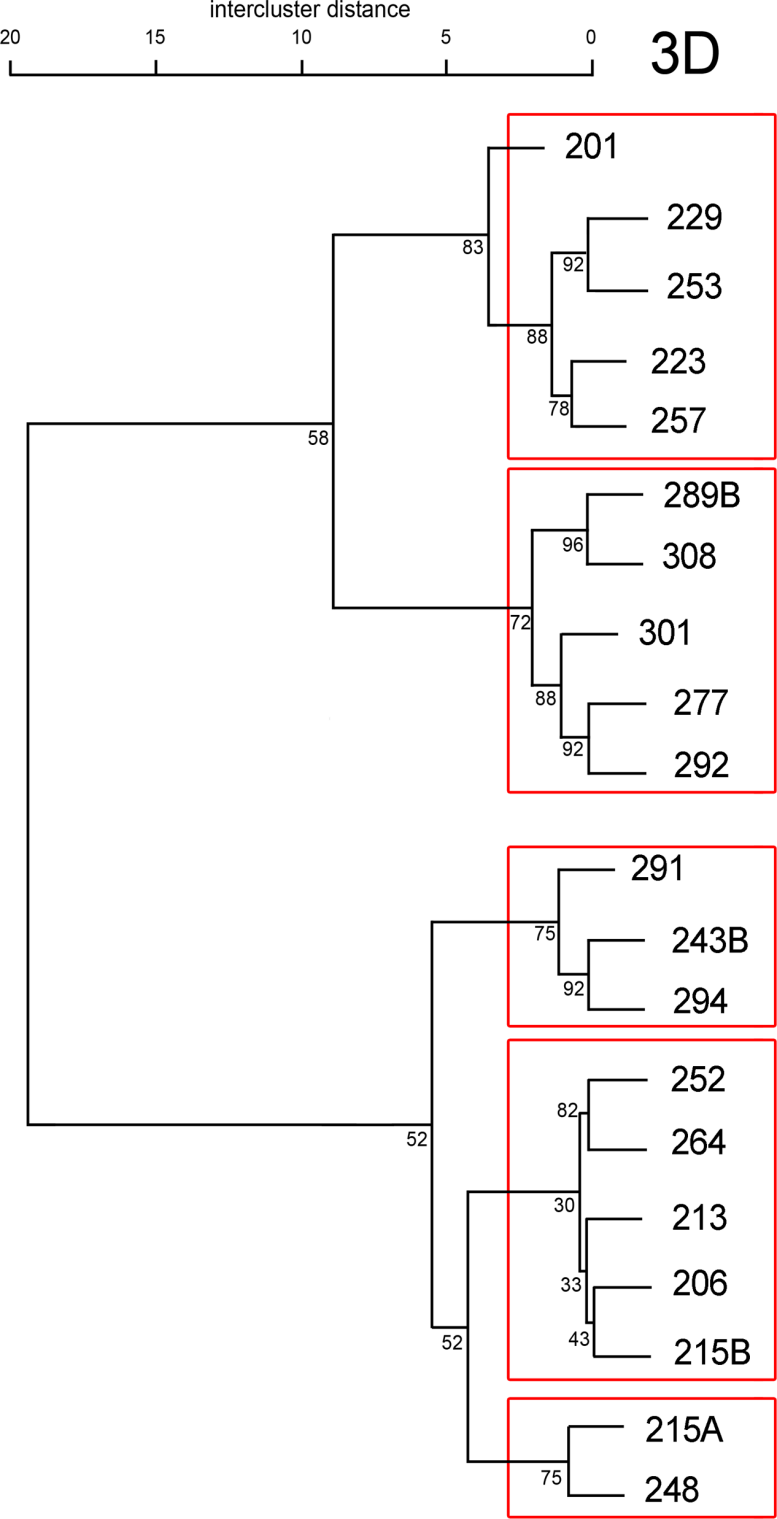
Dendrogram from cluster analysis based on 3D tissue proportions. Bootstrap values are indicated on nodes.

The dendrogram obtained from the application of Ward’s method to 3D variables is presented in **Fig 5**. The intercluster distance values are relatively small, suggesting a high homogeneity within the population. Whichever the dataset used (see **S9 Fig** and **S10 Fig** for cluster analyses performed on 2D variables, on enamel and on dentin components, separately), the results of cluster analysis could be divided into two main clusters. In a large majority, the same groups of individuals are found, especially considering final clusters. At the scale of the necropolis, the dendrogram clusters do not fit well into the spatial organization (see **S11 Fig**), suggesting that biological and spatial distances among individuals may not be strongly associated. However, at a smaller scale, the final clusters identified (**Fig 5**) are mostly supported by bootstrap higher than 90 (e.g., 229–253; 289B-308; 277–292; 243B-294). These subclusters include individuals who share multiple parameters, such as burial structure, orientation and location in the necropolis, position of the head and the body in the burial (**Table 1 and S11 Fig**). Particularly, individuals 206-215B are spatially closed and both are buried in pit with wattling with the same orientation, even if the body of 206 is on the back and that of 215B on the left side, both have the head oriented to the south-east. Also, individuals 215A-248 share pits with alcove and are both buried on the left side with their head to the south.

### EDJ shape

**Fig 6:**
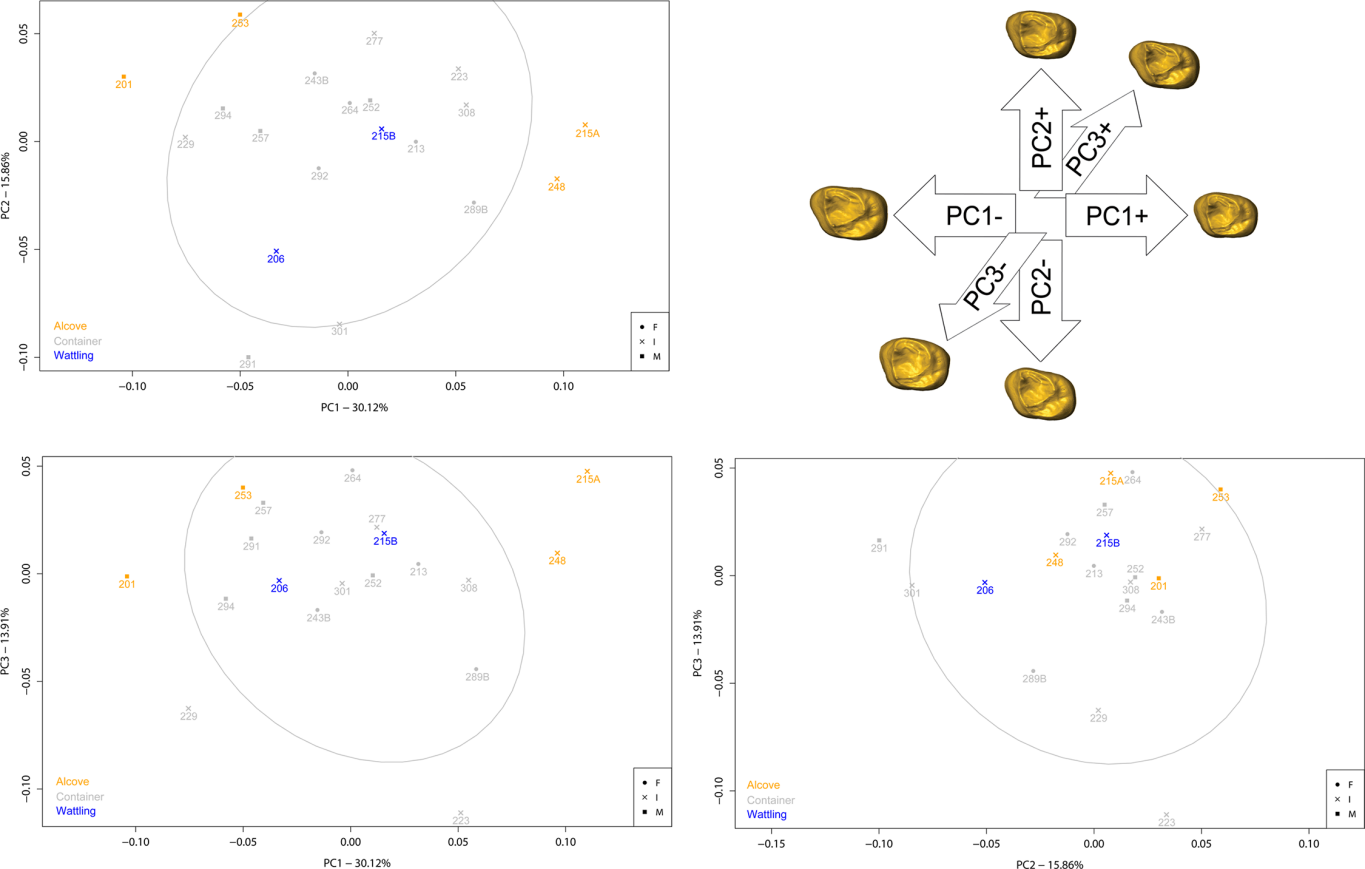
Result of PCA on the EDJ shape coordinates on form space, according to burial structures.

From geometric morphometric analysis, the first three principal components (PC) account for 30.12%, 15.86% and 13.91% of the total form variation of EDJ, respectively (**Fig 6**). PC1 mostly represents allometric size differences observed between males and females (correlation of PC1 and lnCS: r = 0.975), while PC2 and PC3 allow visualization of only size-independent shape variations (correlations of PC2/PC3 and lnCS: r = 0.046 / r = 0.211. respectively). Minor sex differences in overall EDJ shape are observed, but males tend to have bigger EDJ, with higher dentin horn tips and buccolingual lengthening (**S12 Fig**).

Furthermore, individuals buried in a pit with wattling show an EDJ shape that is systematically included in the variability of those buried in pit with container (**Fig 6**). On the contrary, allometry particularly affects individuals buried in pit with alcove which are outside the variability observed for other individuals along PC1 (**Fig 6**). While males 201 and 253 possess the biggest EDJ and individuals 215A and 248 the smallest, these four individuals share a similar condition, illustrated by their position along PC3. Indeed, they present a reduction of the height of the distolingual dentin horn tip (hypocone) compared to the height of other horn tips.

**Fig 7:**
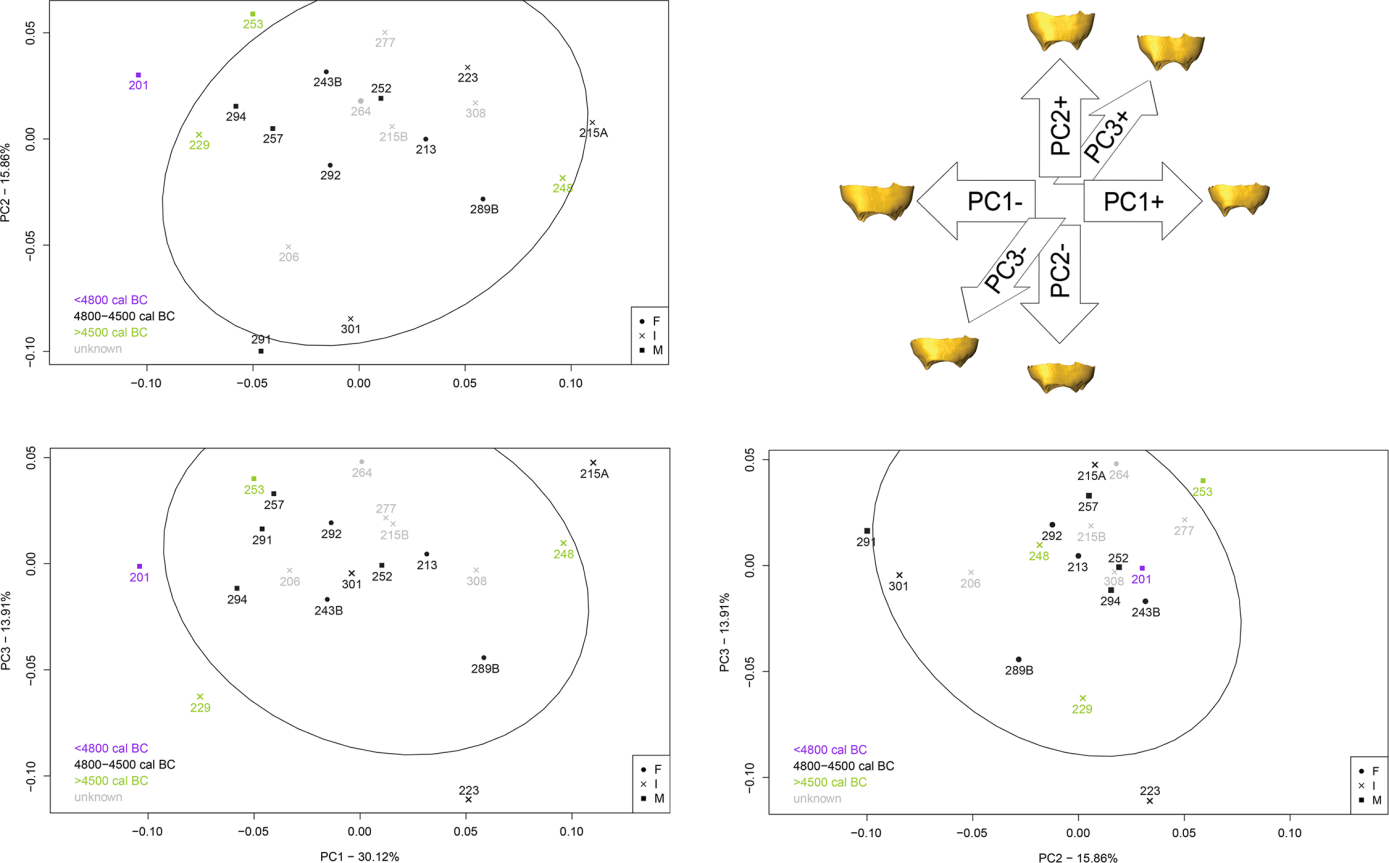
Result of PCA on the EDJ shape coordinates on form space, according to occupation phases.

Individuals from early (A) and recent (C) phases have EDJ shapes that fall at the extreme variation shown by the individuals from the principal phase (**Fig 7**). In particular, individual 201 from the early phase possesses an EDJ shape that sets him apart. As a whole, individuals from early and recent phases, and those buried in alcove seems to be distinguishable by their crown tissue proportions and EDJ shape, distinguishes them from the other individuals buried in the necropolis of Gurgy.

## Discussion

In this study, crown tissue proportions, thickness and distribution of enamel, and EDJ shape have been used to explore phenotypic variations between the Neolithic individuals buried in the necropolis of Gurgy.

- Do phenotypic distances obtained from the study of morphological and metrical variation of internal tooth structure are a reliable proxy to track groups sharing similar chronocultural and burial practices?

While a relative homogeneity has been found in these variables, and particularly in enamel thickness topography, differences from internal tooth structure analysis could be correlated with burial and chronocultural parameters. Individuals buried in pits with alcove show subtle differences in their internal tooth structure from individuals found in pits with container and wattling. Moreover, individuals from early and recent phases of the necropolis could be distinguished from those of the principal phase from their crown tissue proportions and EDJ shape. Moreover, it is noteworthy that pits with alcove are located at the south-west periphery of the necropolis area (see **Fig 1**). They were continuously used during the occupation of the necropolis, which started at the end of Early Neolithic period and ended during the Middle Neolithic period [112]. However, the only structure consisting of an alcove that is similar to those observed during the *Rubané Récent du Bassin Parisien (RRBP)* is the burial of individual 201 [150]. The RRBP is the latest expansion of the *Linearbandkeramik-derived* culture in Western Europe [151,152]. Moreover, paleogenetic data showed that both haplogroups inherited from hunter-gatherers and farmers are found in individuals buried in alcove [114]. The present data suggest that these individuals may have shared a cultural and phenotypical heritage from Early Neolithic. Also, despite the geographical location of the Neolithic necropolis of Gurgy at the confluence of the two waves of neolithization [111] and paleogenetic evidence of individuals derived from both waves [114], the funerary practices are relatively homogeneous at Gurgy [112], especially compared to the diversity that has been observed in the Paris Basin during Early/Middle Neolithic [115,153–156]. Moreover, data from isotopic analysis [117], enamel thickness topography and occlusal wear patterns [93] show an important homogeneity in the Neolithic population of Gurgy. In this context, the relative cultural and phenotypical distinctions of individuals buried in an alcove may more likely be in relation to inherited patterns from Ancient Neolithic groups.

- Is one of these internal parameters more relevant to discuss interindividual variability?

Considering the subtle differences found at the intrasite scale, none of the internal parameters proved to be more relevant, it was the whole crown analysis that allowed to discuss differences linked to chronocultural variations and burial structures. However, the dentin component should be interpreted cautiously. In the necropolis of Gurgy, males have a greater quantity of crown tissues than females, but only the dentin volume differs significantly between sexes. Overall dimensions of the crown and dentin volume must be considered in the interpretation of the results. Also, the EDJs of males tend to be bigger than those of females, with higher dentin horn tips and buccolingual lengthening. These results are consistent with sexual dimorphism that has already been shown for internal crown structure, with men having higher enamel volume, dentin volume and EDJ area than women [109,110,157]. In this context, previous studies suggested that sexual dimorphism in external crown dimensions could be linked with a higher production of dentin in males compared to females [157–160].

- Can causes of dental variations be suggested?

While individuals buried in alcove show a size‐related variation in internal crown proportions and morphology which differentiate males and females, both tend to share an identical size-independent EDJ shape trend. Indeed, it seems that the individuals buried in pits with alcove share a reduction in the height of their distolingual dentin horn tip, corresponding to the hypocone. This functional cusp [161,162] which is the last to form during dental development [96,103,104] shows higher variability. This could be consistent with previous studies which showed that distal cusps are more plastic to environmental stress [163,164]. Besides, according to the patterning cascade model of cusp development [107], the location, size, and shape of the later-developing cusps are configured by the characteristics of the first-forming cusps [103]. This model predicts that small initial differences in cusp spacing will have cumulative effects on later-developing cusps during the morphogenetic process [107]. Also, while asymmetry of enamel thickness topography is systematically shown between the functional and the non-functional cusps of UM2 at Gurgy [93], the individuals in alcove share a slightly more homogenous pattern of enamel thickness distribution than that of the other individuals. While enamel thickness has been demonstrated to be an evolutionary plastic trait, selectively responsive to functionally-related dietary changes and wear [20,61,78,93,165], thick enamel has been shown to be a homoplastic trait [61]. Functionally-related adaptive changes of enamel have been demonstrated, notably enamel thickness is an evolutionary plastic trait selectively responsive to dietary changes, tooth fracture and occlusal wear [20,61,78,93,165]. Moreover, the distolingual cusp was expected to be the most sensitive to functional changes [93]. Thus, from the preliminary samples used in this study, differences at phenotypical and cultural levels are found for these Neolithic individuals. The balance between environmental, phylogenetic, developmental and functional aspects is hard to evaluate, and any of these aspects can be suggested as possible underlying cause for the origin of phenotypical differences shared by these individuals buried in alcove.

## Conclusions

In this study of the Neolithic individuals from Gurgy, morphological and metric parameters such as crown tissue proportions, thickness and distribution of enamel, and EDJ shape, were assessed in a whole crown perspective, in order to finely quantify size and shape variations in a microevolutionary context. The results suggest that the internal tooth structure may be a reliable proxy to track groups sharing similar chronocultural and burial practices. Indeed, from the non-invasive characterization of their internal tooth structure, individuals buried in alcove have been distinguished from those buried in other structures, and underlying factors and causes of these dental variations have been discussed. The internal tooth structure could be used to discuss interindividual phenotypic variation within and between burial grounds, as well as to assess environmental, phylogenetic, developmental and/or functional underlying causes of these phenotypic variations. With adapted methods and templates designed to finely characterize variations at microevolutionary scales, further studies of teeth from osteological reference collections will elucidate to which extent these factors may be tracked in archeological samples.

## Supporting Information

S1 Fig Images of the three burial structures: pit with container (left), pit with wattling (middle), and pit with alcove (right).
(TIF)

S2 Fig Multiple plot of radiocarbon ages available for the sampled individuals. (TIF)

S3 Fig Virtual buccolingual cross-section through the dentin horn tips of the mesial cusps of upper second molar, surface and linear variables (left), and standardized enamel thickness measured on the buccal aspect (right).
(TIF)

S4 Fig 3D surface models of upper second molar (a); dental tissues in transparence (b) (enamel in white, dentin in yellow, pulp in orange) with position of the cervical plane for virtual isolation of the crown; and resulting measured volumes: total crown volume (c), enamel volume (d), coronal dentin volume (e).
(TIF)

S5 Fig EDJ template. Landmarks are represented in black spheres, curve semilandmarks in grey spheres, and surface semilandmarks in white spheres.
(TIF)

S6 Fig Plot of c against b (left) and plot of b against AET2D (right) according to the sex of individuals.
(TIF)

S7 Fig Comparative morphology of each UM2 crown. Reconstructions of outer enamel surface (OES), enamel-dentin junction (EDJ), dental tissue proportions (DTP) with superposition of enamel and dentin in transparence, and cartography of enamel thickness (ET) in occlusal (O), mesial (M), distal (D), buccal (B) and lingual (L) views.
(PDF)

S8 Fig Plot of Evol against DPvol (left) and plot of DPvol against AET3D (right) according to the sex of individuals.
(TIF)

S9 Fig Dendrogram from cluster analysis based on 2D tissue proportions. Bootstrap values are indicated on nodes.
(TIF)

S10 Fig Cluster analysis performed on enamel component only (Evol and c, left) and dentin component only (DPvol and b, right).
(TIF)

S11 Fig Necropolis map of Gurgy with details of cultural parameters for individuals clustered according to the dendrogram obtained from 3D tissue proportions. (TIF)

S12 Fig ACP on EDJ according to the sex of individuals. (TIF)
