# Supplementary figures and images for: Internal Tooth Structure and Burial Practices: Insights into the Neolithic Necropolis of Gurgy (France, 5100-4000 cal. BC)
Source: PLoS One. 2016 Jul 22;11(7):e0159688. doi: 10.1371/journal.pone.0159688 (PMC4957824; doi:10.1371/journal.pone.0159688)

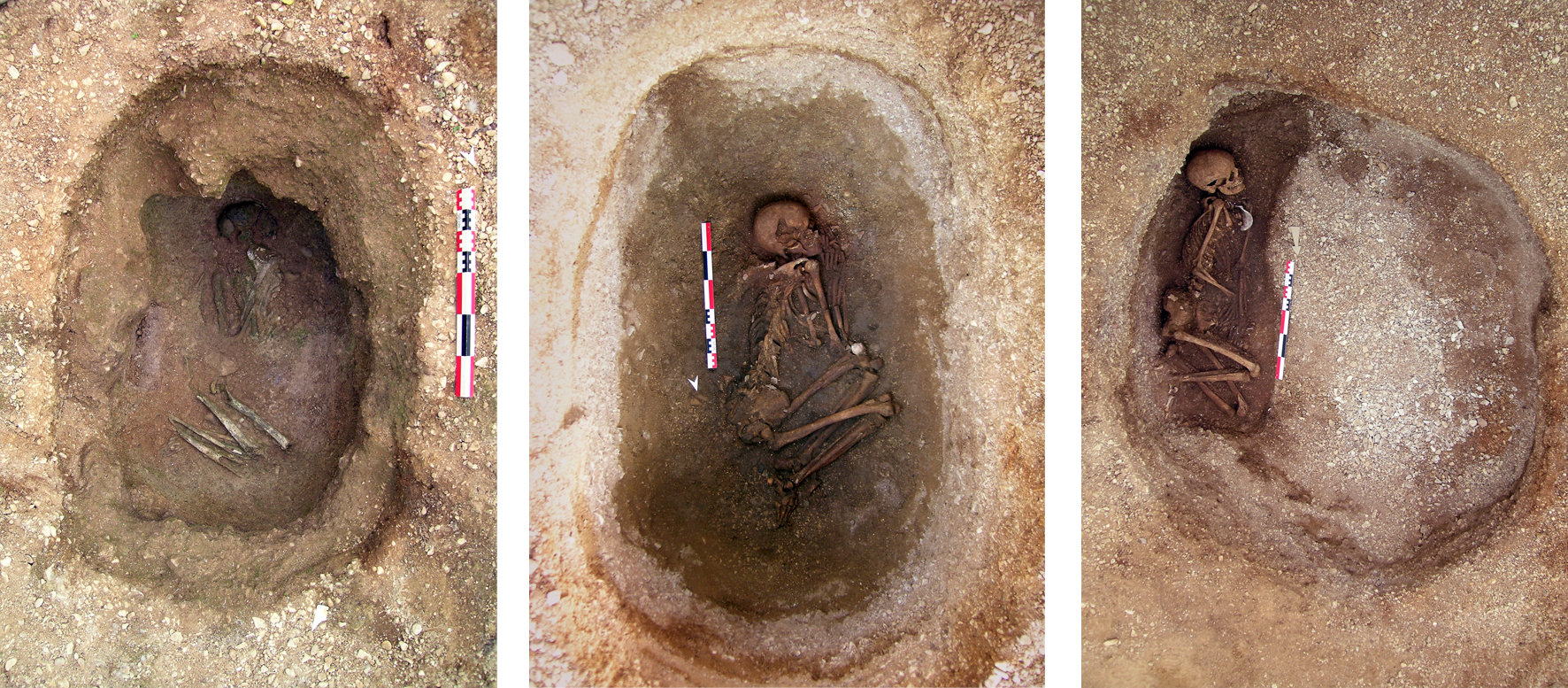

Supplement: S1 Fig — Images of the three burial structures: pit with container (left), pit with wattling (middle), and pit with alcove (right). (TIF) [file pone.0159688.s001.tif]

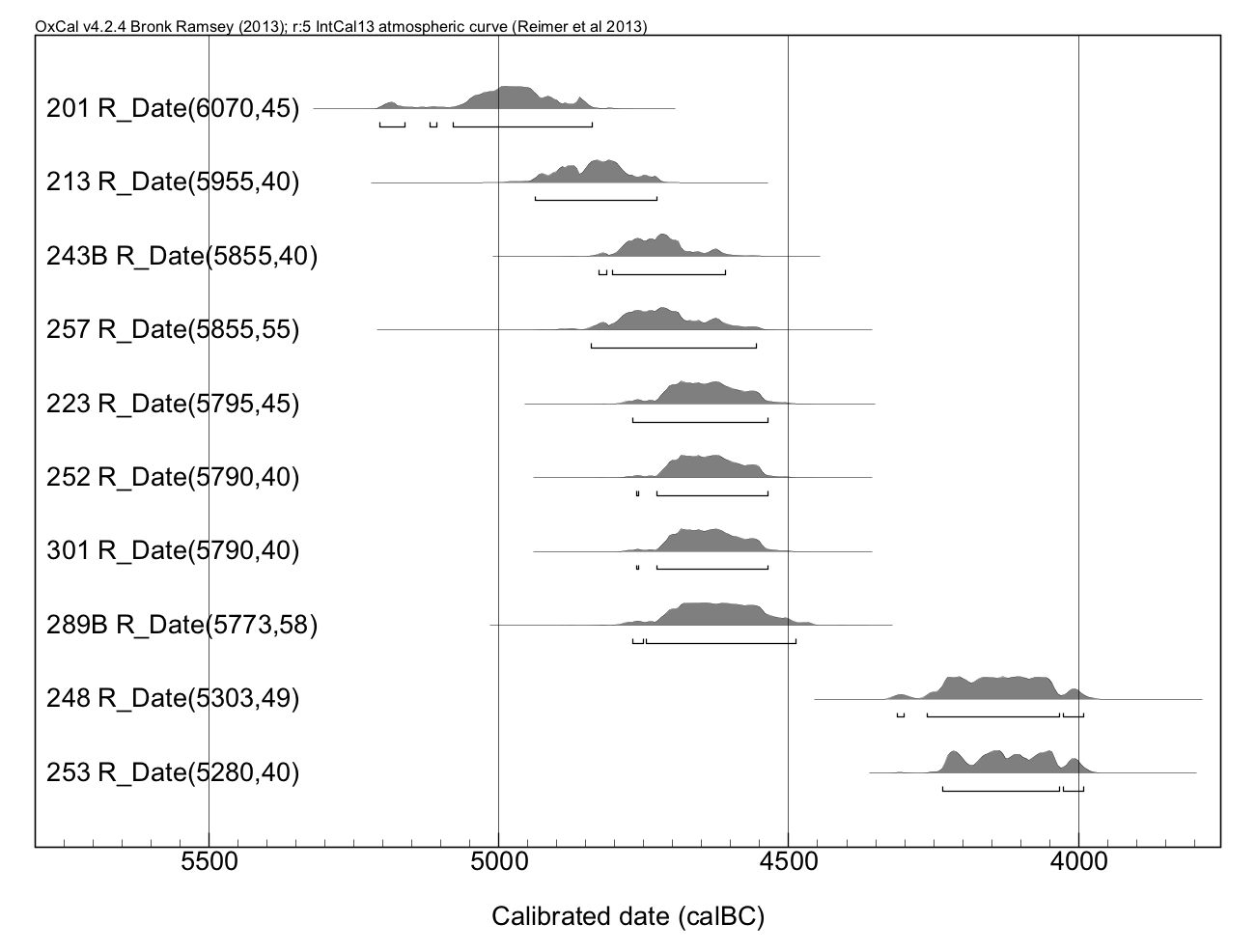

Supplement: S2 Fig — (TIF) [file pone.0159688.s002.tif]

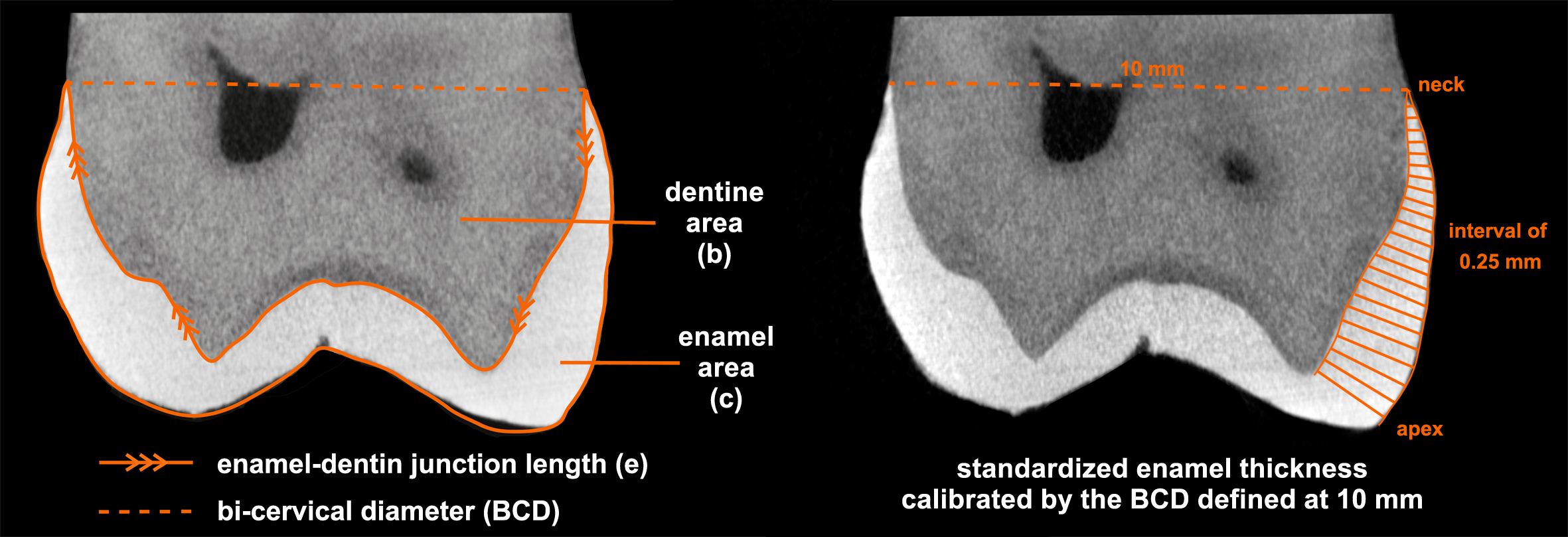

Supplement: S3 Fig — Virtual buccolingual cross-section through the dentin horn tips of the mesial cusps of upper second molar, surface and linear variables (left), and standardized enamel thickness measured on the buccal aspect (right). (TIF) [file pone.0159688.s003.tif]

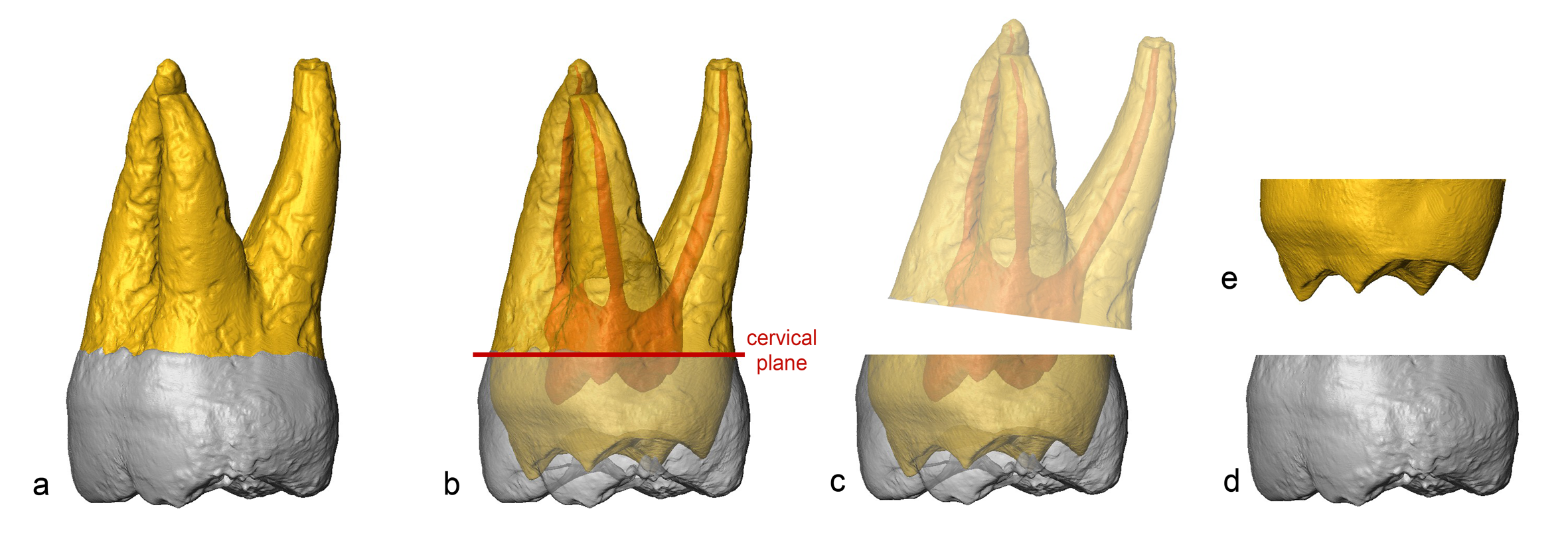

Supplement: S4 Fig — 3D surface models of upper second molar (a); dental tissues in transparence (b) (enamel in white, dentin in yellow, pulp in orange) with position of the cervical plane for virtual isolation of the crown; and resulting measured volumes: total crown volume (c), enamel volume (d), coronal dentin volume (e). (TIF) [file pone.0159688.s004.tif]

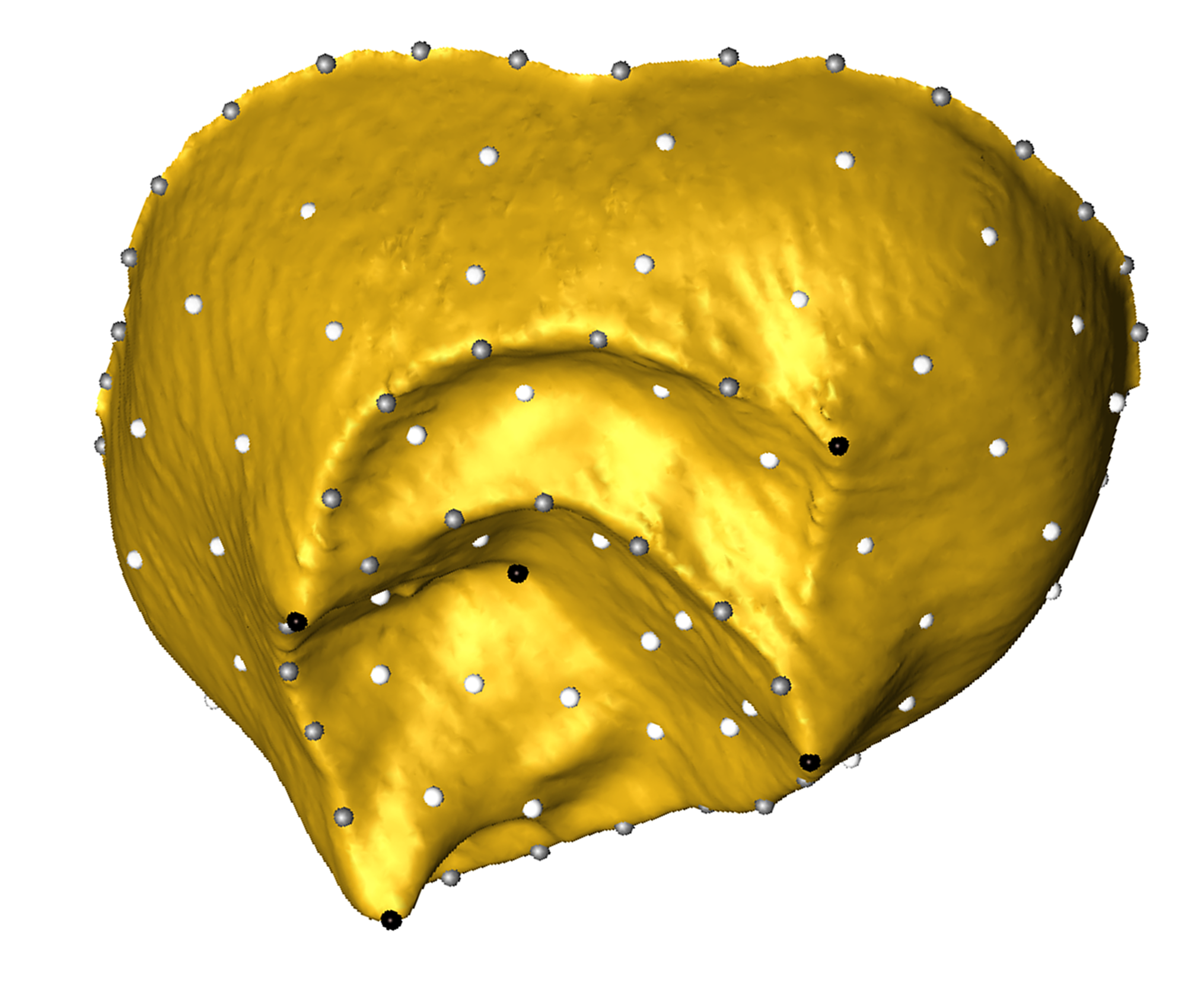

Supplement: S5 Fig — Landmarks are represented in black spheres, curve semilandmarks in grey spheres, and surface semilandmarks in white spheres. (TIF) [file pone.0159688.s005.tif]

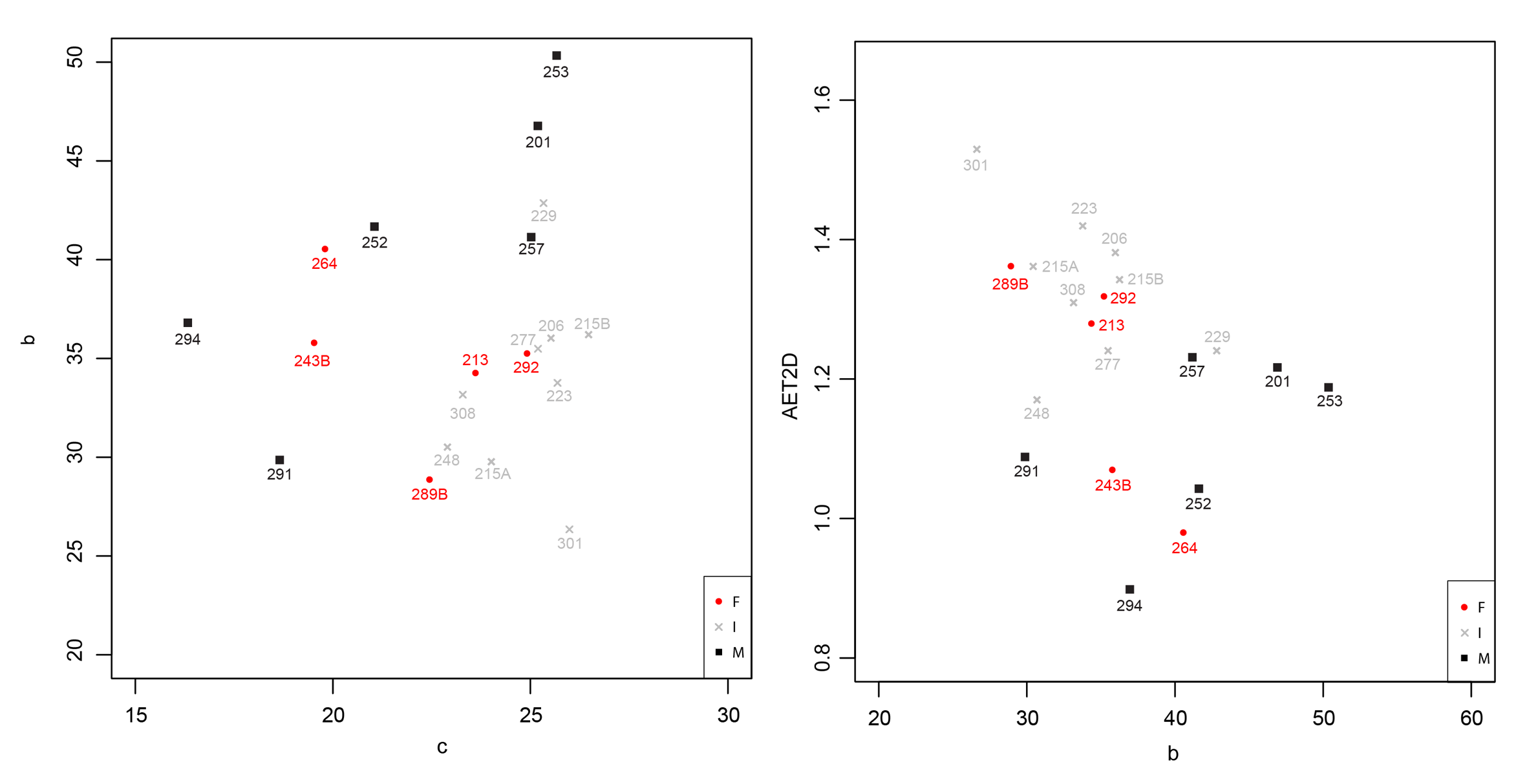

Supplement: S6 Fig — Plot of c against b (left) and plot of b against AET2D (right) according to the sex of individuals. (TIF) [file pone.0159688.s006.tif]

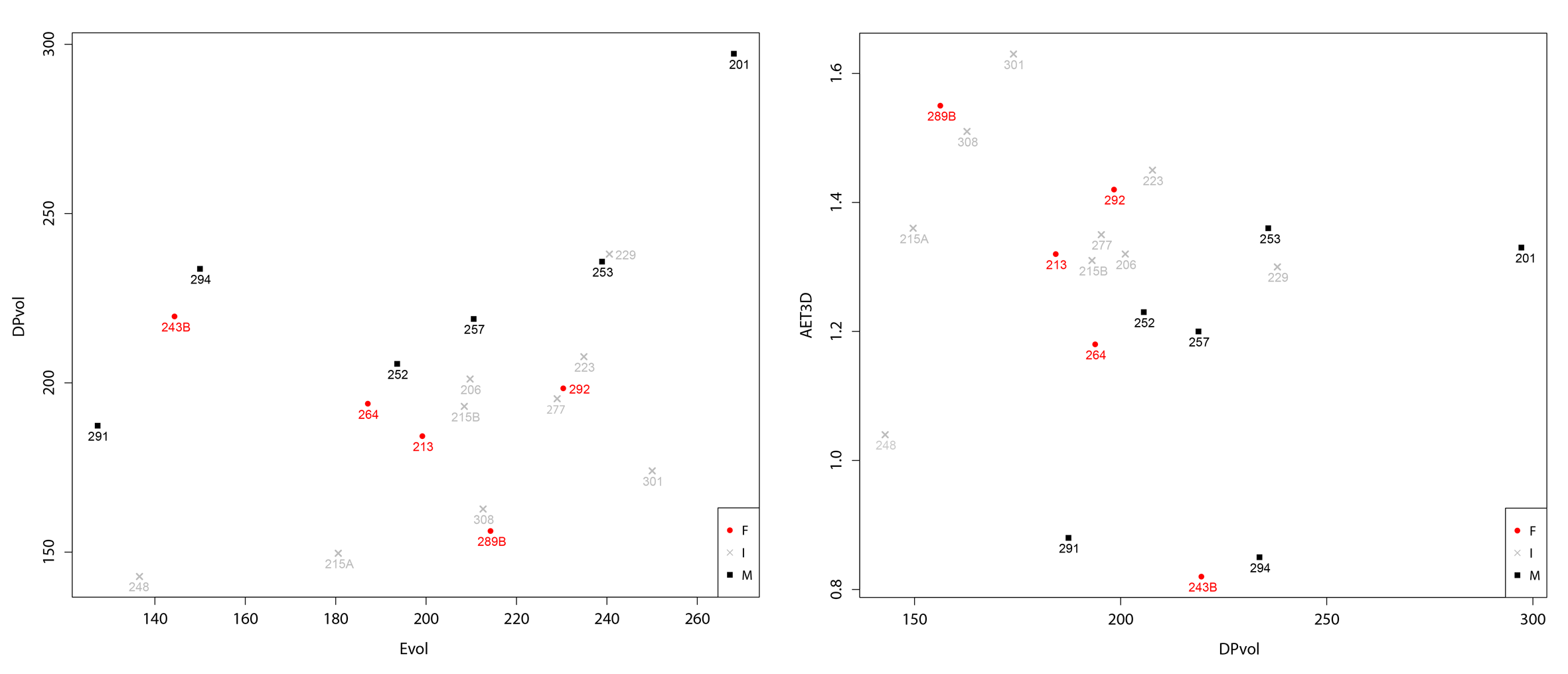

Supplement: S8 Fig — Plot of Evol against DPvol (left) and plot of DPvol against AET3D (right) according to the sex of individuals. (TIF) [file pone.0159688.s008.tif]

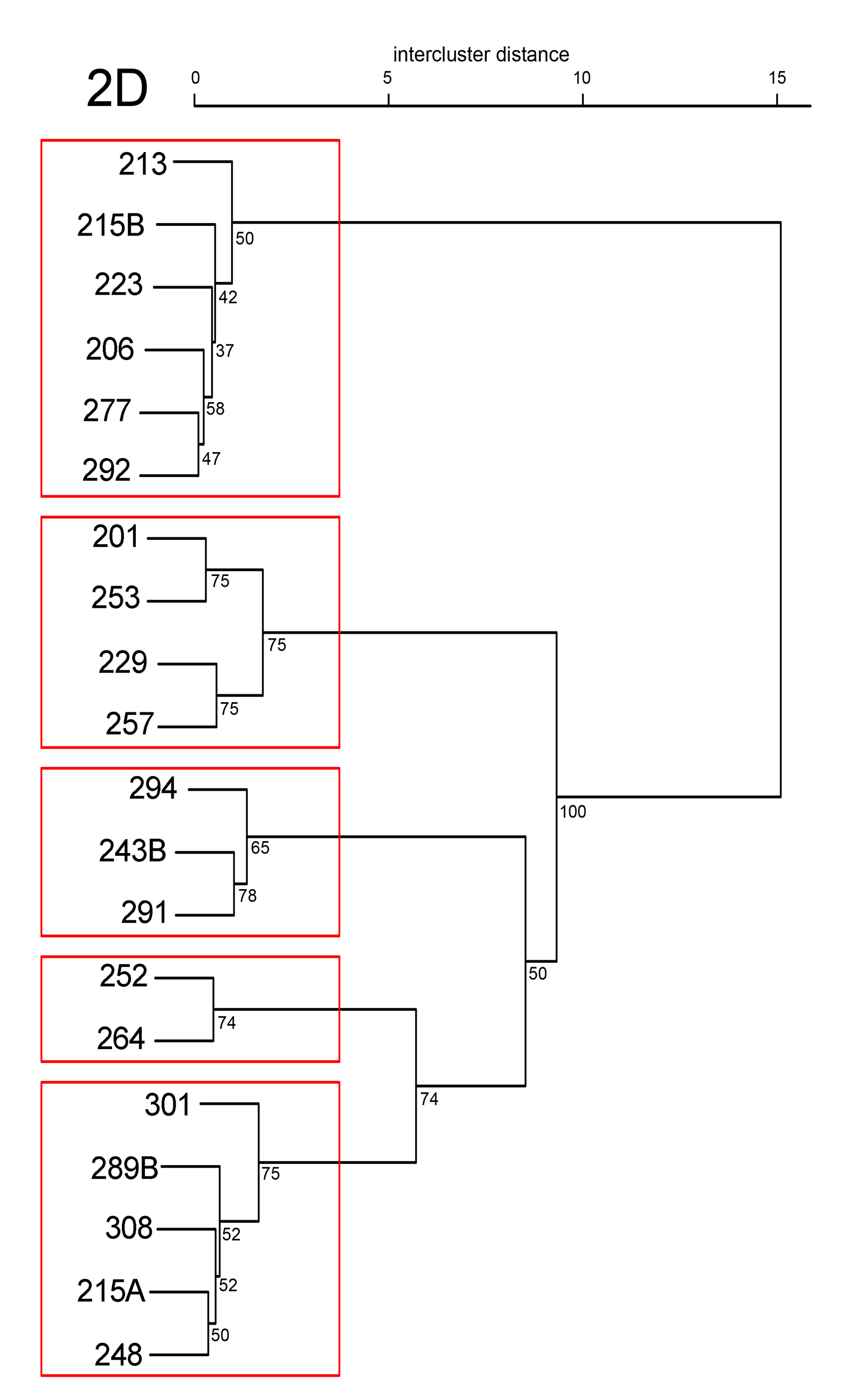

Supplement: S9 Fig — Bootstrap values are indicated on nodes. (TIF) [file pone.0159688.s009.tif]

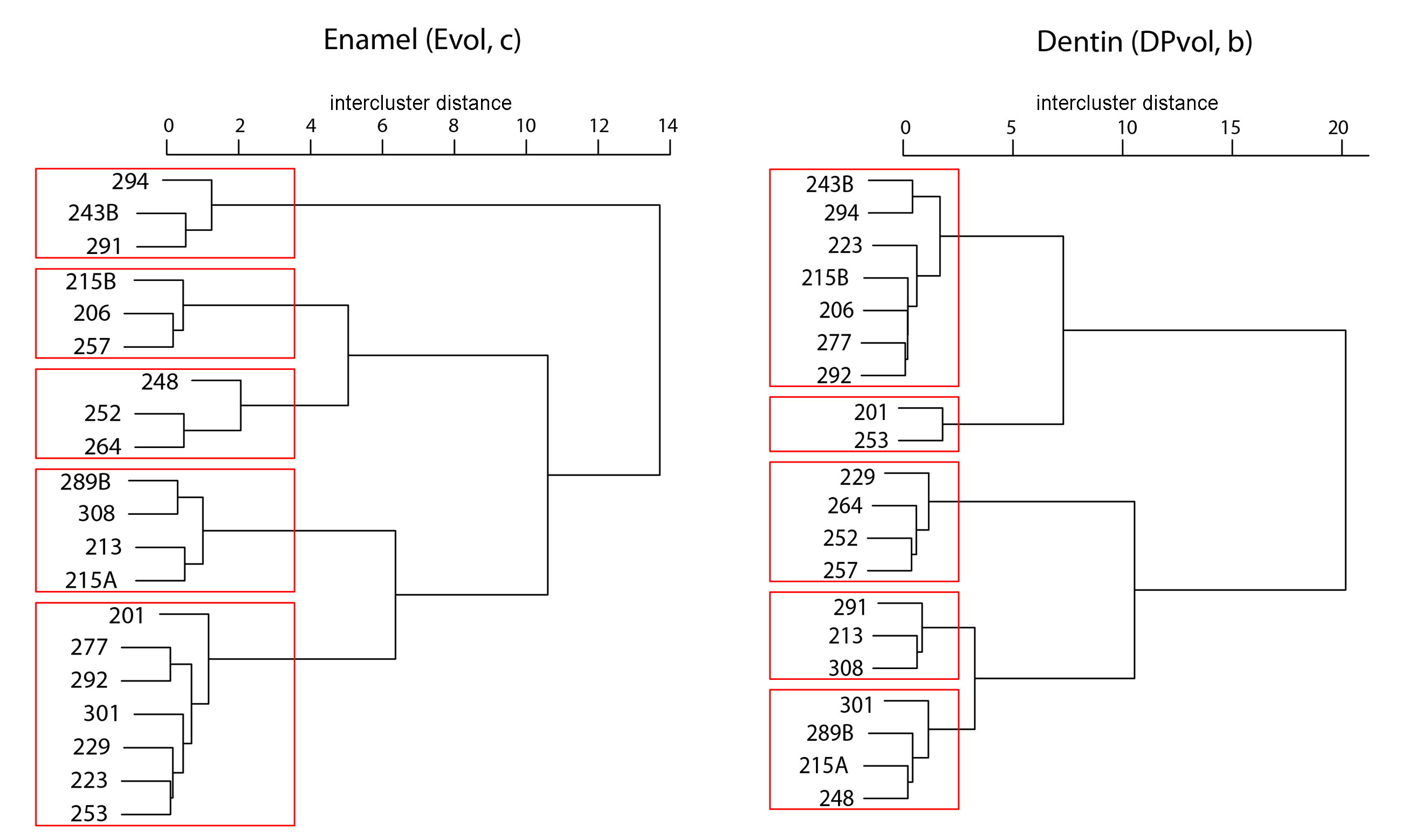

Supplement: S10 Fig — Cluster analysis performed on enamel component only (Evol and c, left) and dentin component only (DPvol and b, right). (TIF) [file pone.0159688.s010.tif]

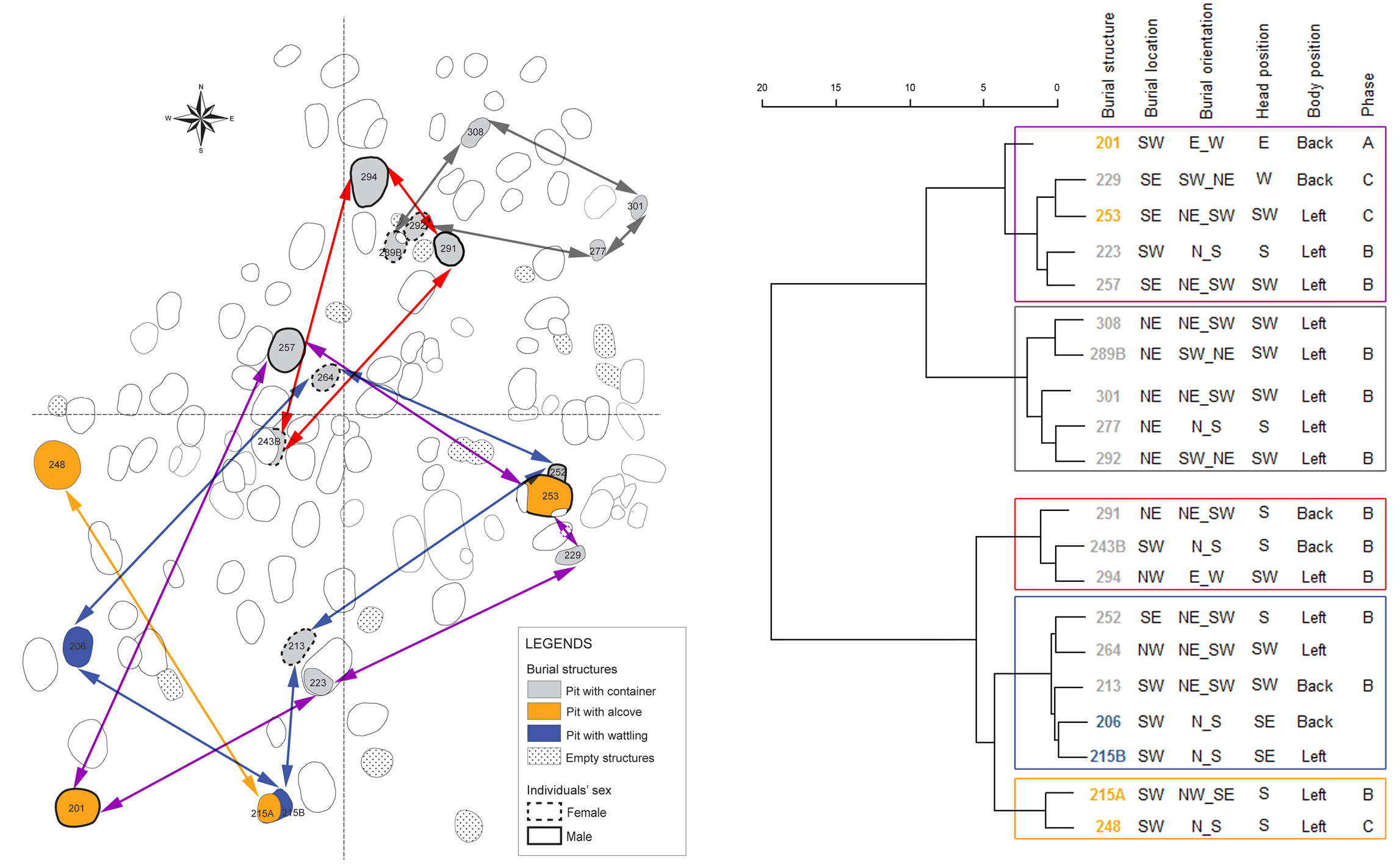

Supplement: S11 Fig — (TIF) [file pone.0159688.s011.tif]

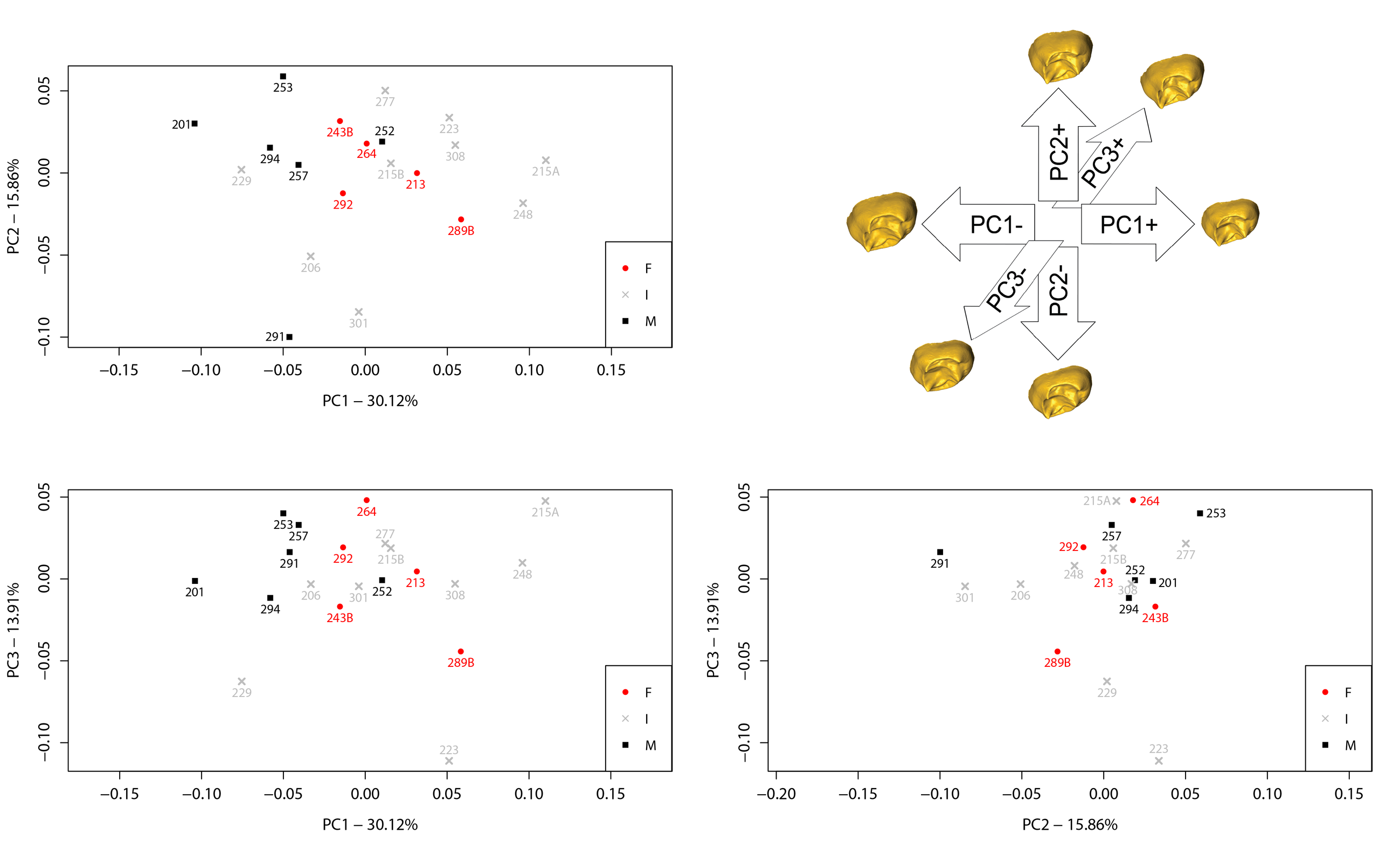

Supplement: S12 Fig — (TIF) [file pone.0159688.s012.tif]
