# Supplementary material for: Internal Tooth Structure and Burial Practices: Insights into the Neolithic Necropolis of Gurgy (France, 5100-4000 cal. BC)
Source: PLoS One. 2016 Jul 22;11(7):e0159688. doi: 10.1371/journal.pone.0159688 (PMC4957824; doi:10.1371/journal.pone.0159688)
Supplement: S7 Fig — Reconstructions of outer enamel surface (OES), enamel-dentin junction (EDJ), dental tissue proportions (DTP) with superposition of enamel and dentin in transparence, and cartography of enamel thickness (ET) in occlusal (O), mesial (M), distal (D), buccal (B) and lingual (L) views. (PDF) [file pone.0159688.s007.pdf]

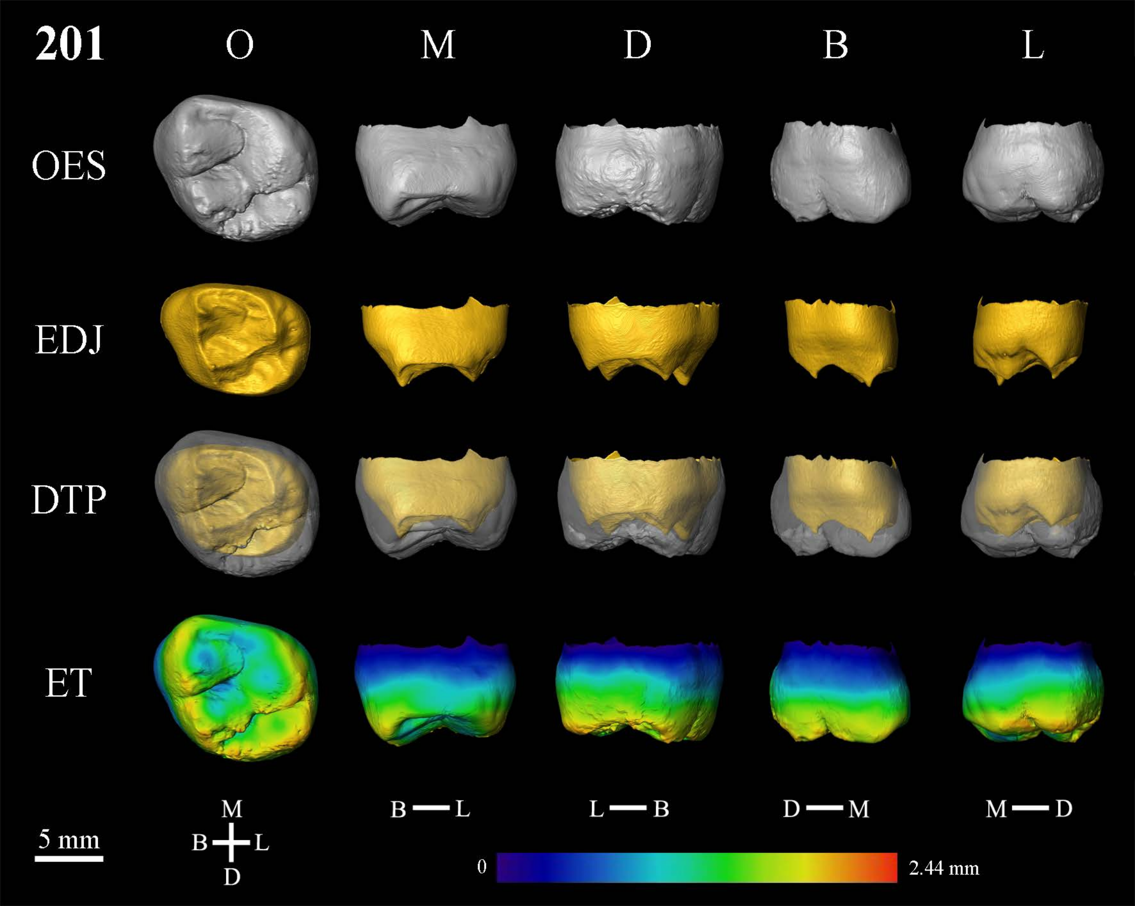
201
O
M
D
B
L
OES
EDJ
DTP
ET
M
B L
D
B — L
L — B
D — M
M — D
5 mm
0
2.44 mm

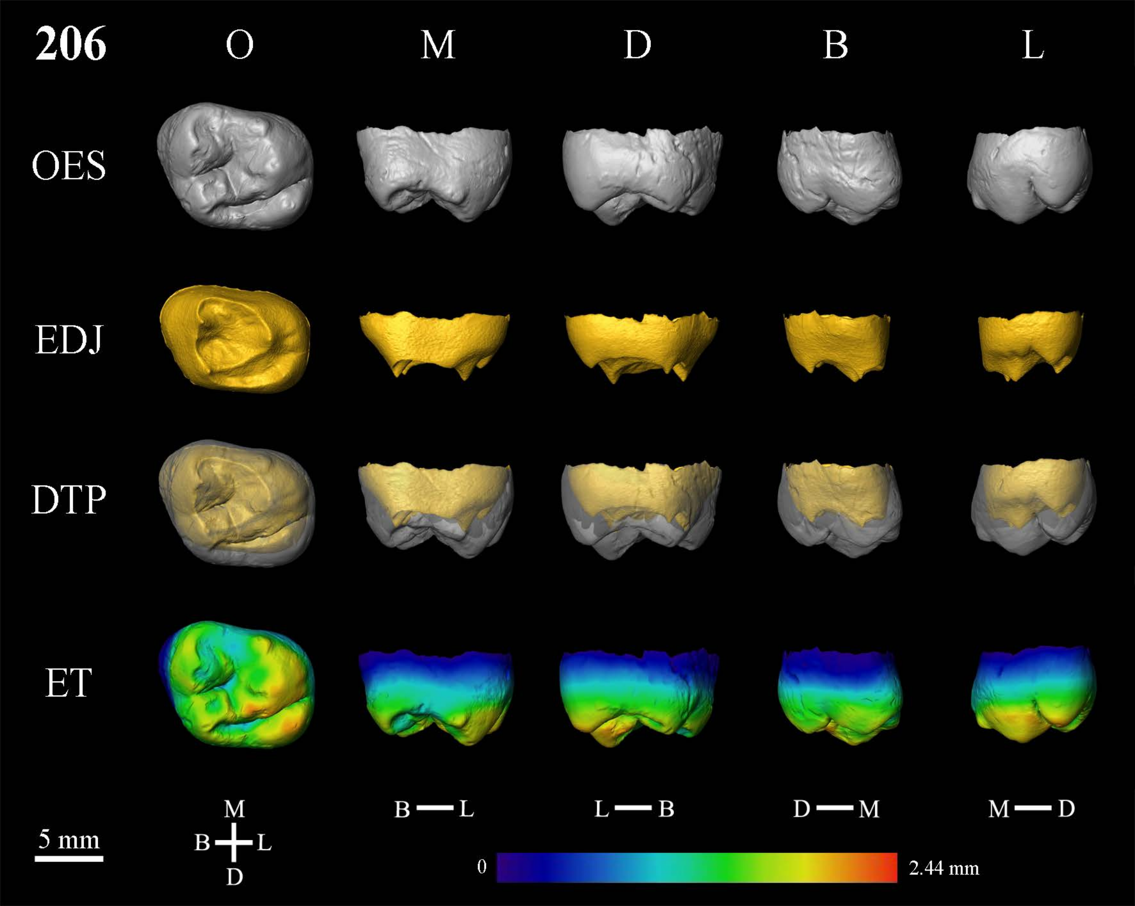
206
O
M
D
B
L
OES
EDJ
DTP
ET
M
B
L
D
B L
L B
D M
M D
5 mm
0
2.44 mm

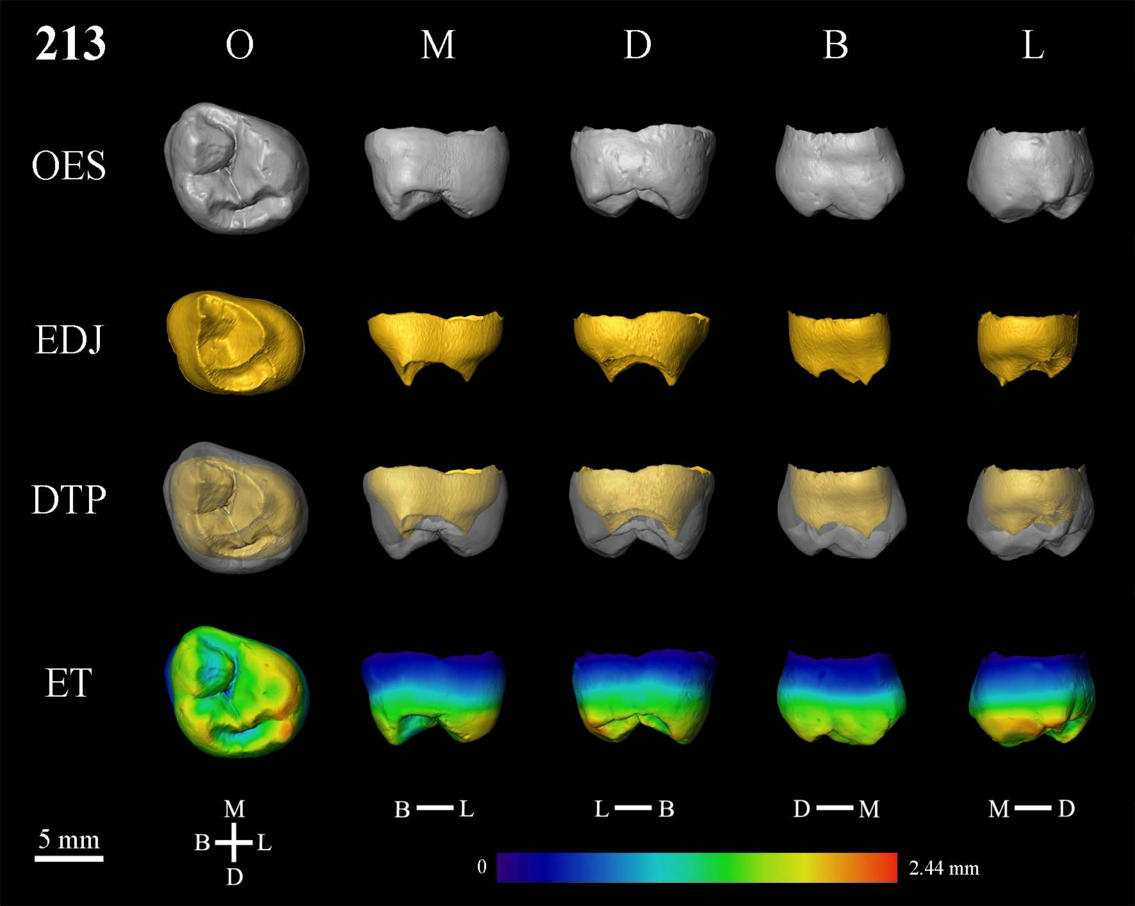
213
O
M
D
B
L
OES
EDJ
DTP
ET
M
B
L
D
B — L
L — B
D — M
M — D
5 mm
0
2.44 mm

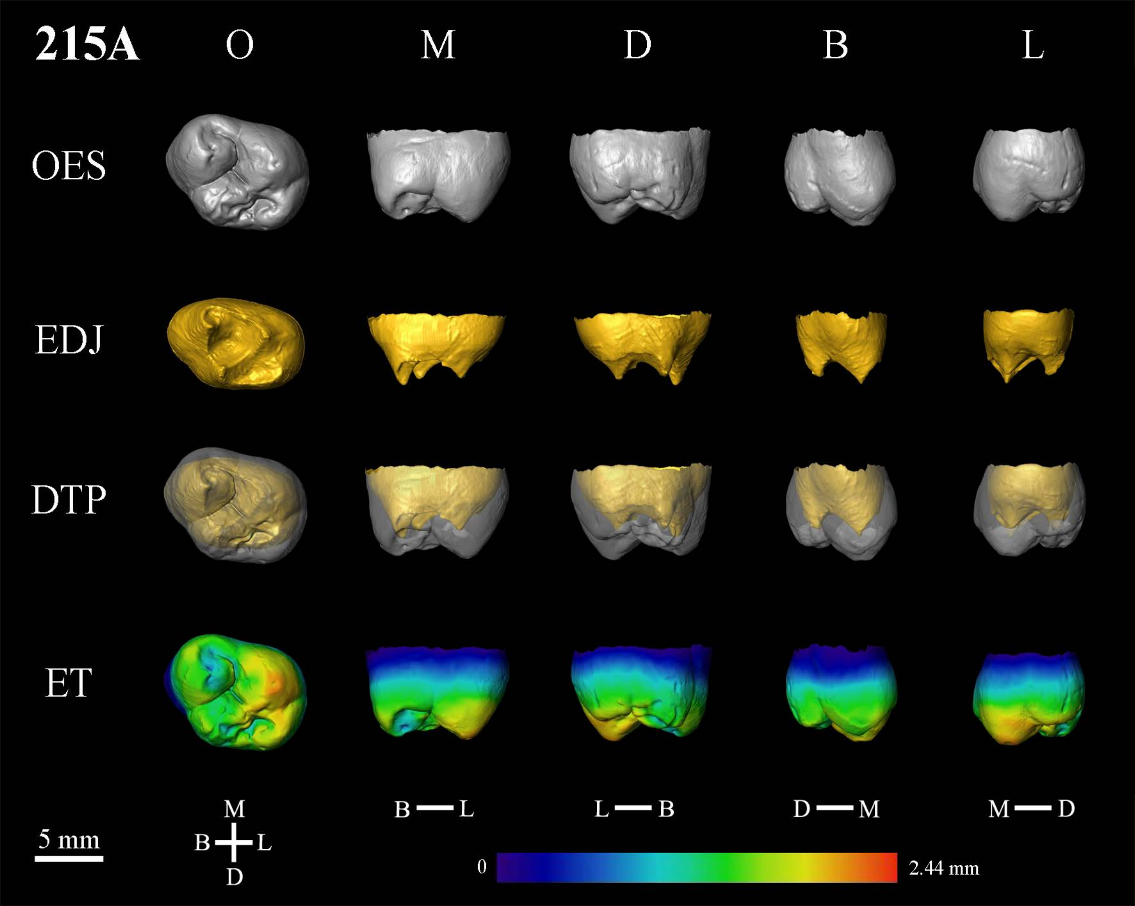
215A
O
M
D
B
L
OES
EDJ
DTP
ET
M
B
L
D
B — L
L — B
D — M
M — D
5 mm
0
2.44 mm

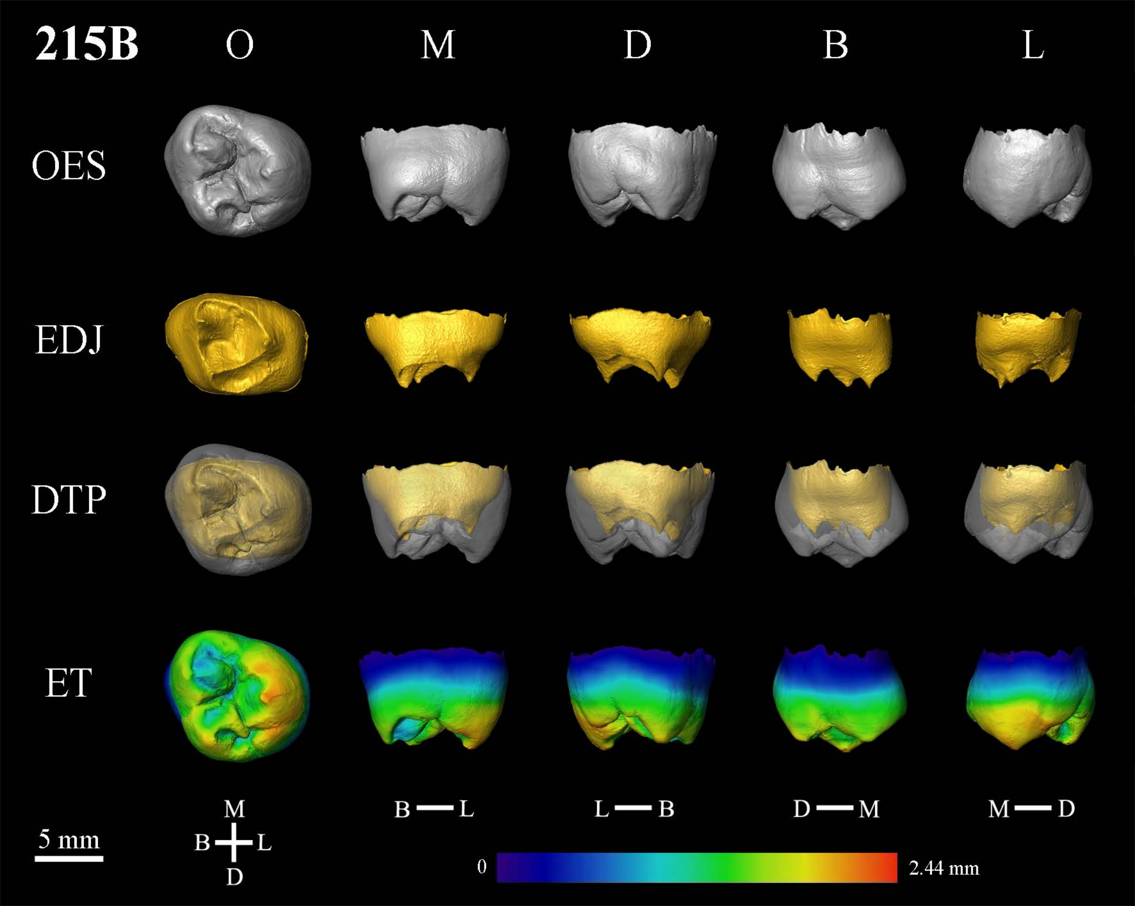

215B
O
M
D
B
L
OES
EDJ
DTP
ET
M
B L
D
B L
L B
D M
M D
5 mm
0
2.44 mm

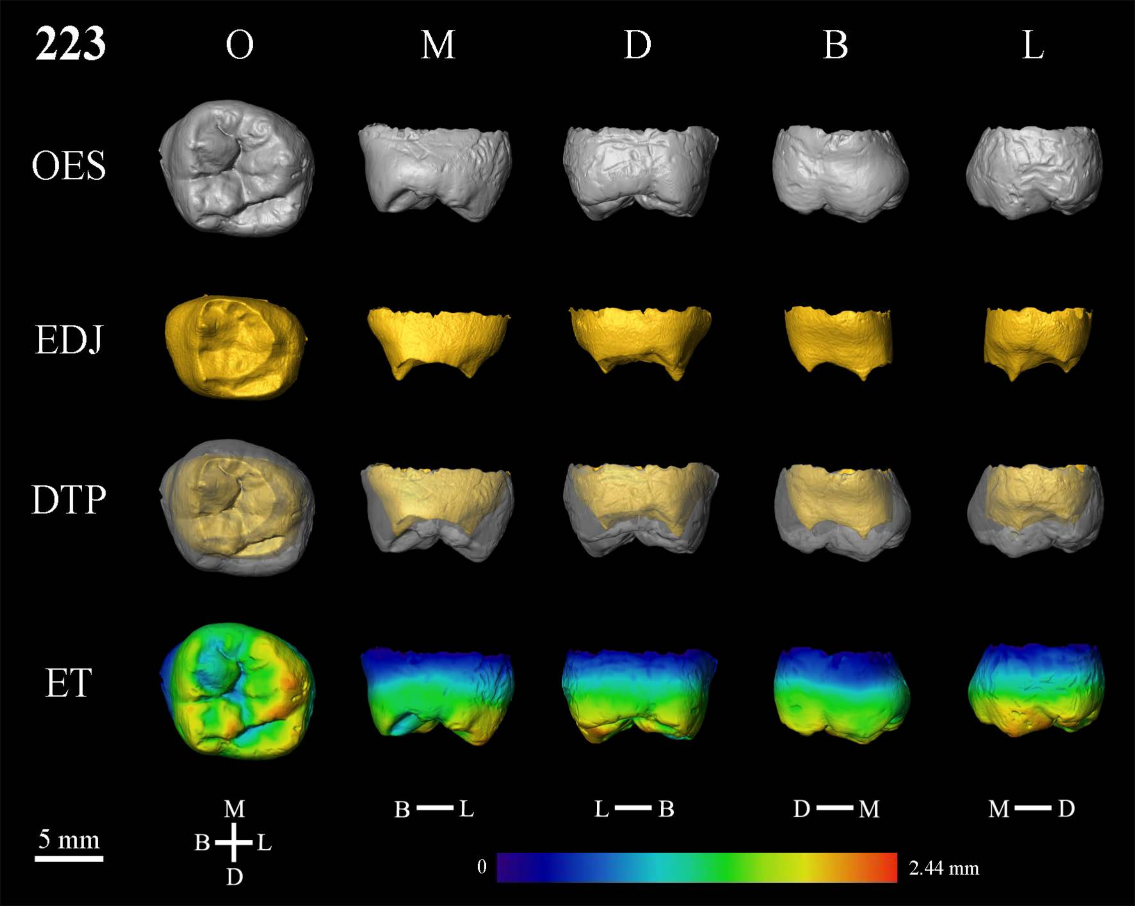
223
O
M
D
B
L
OES
EDJ
DTP
ET
M
B L
D
B L
L B
D M
M D
5 mm
0
2.44 mm

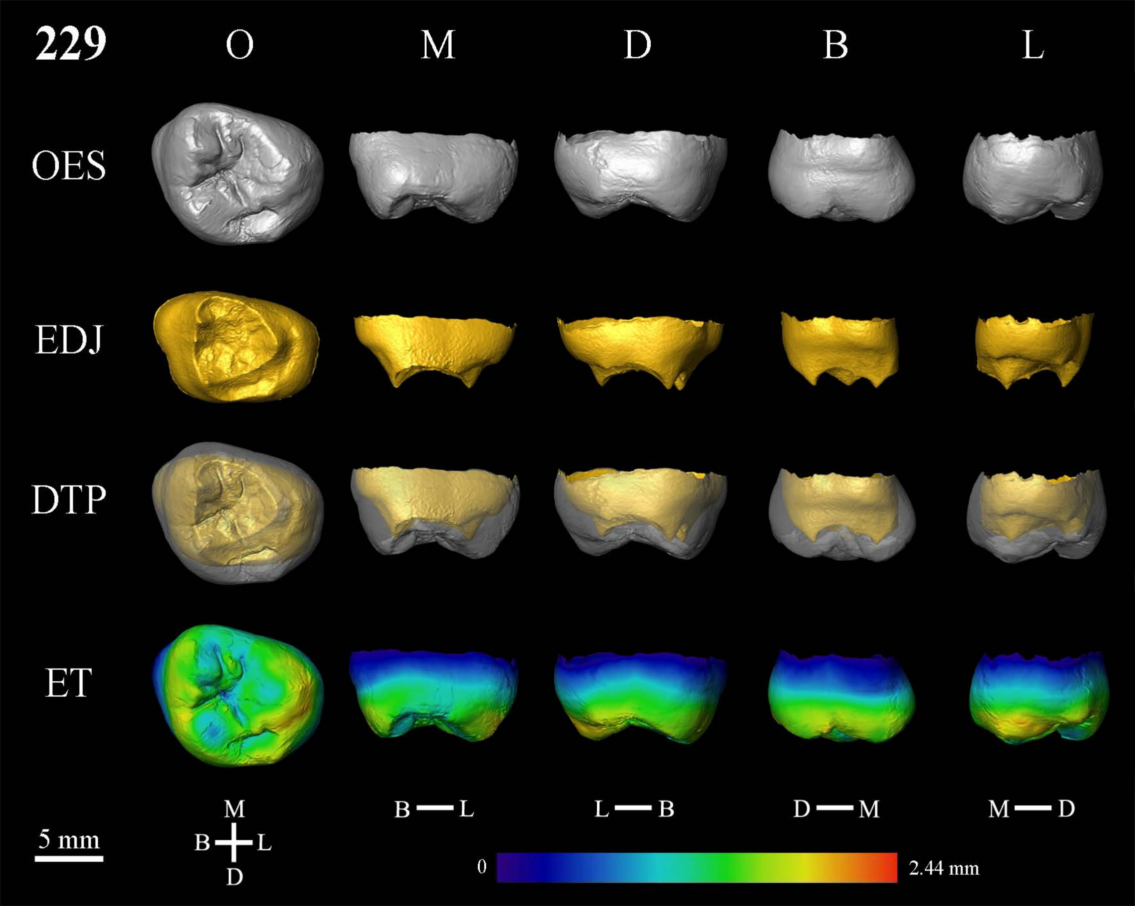

229
O
M
D
B
L
OES
EDJ
DTP
ET
M
B
L
D
B — L
L — B
D — M
M — D
5 mm
0
2.44 mm

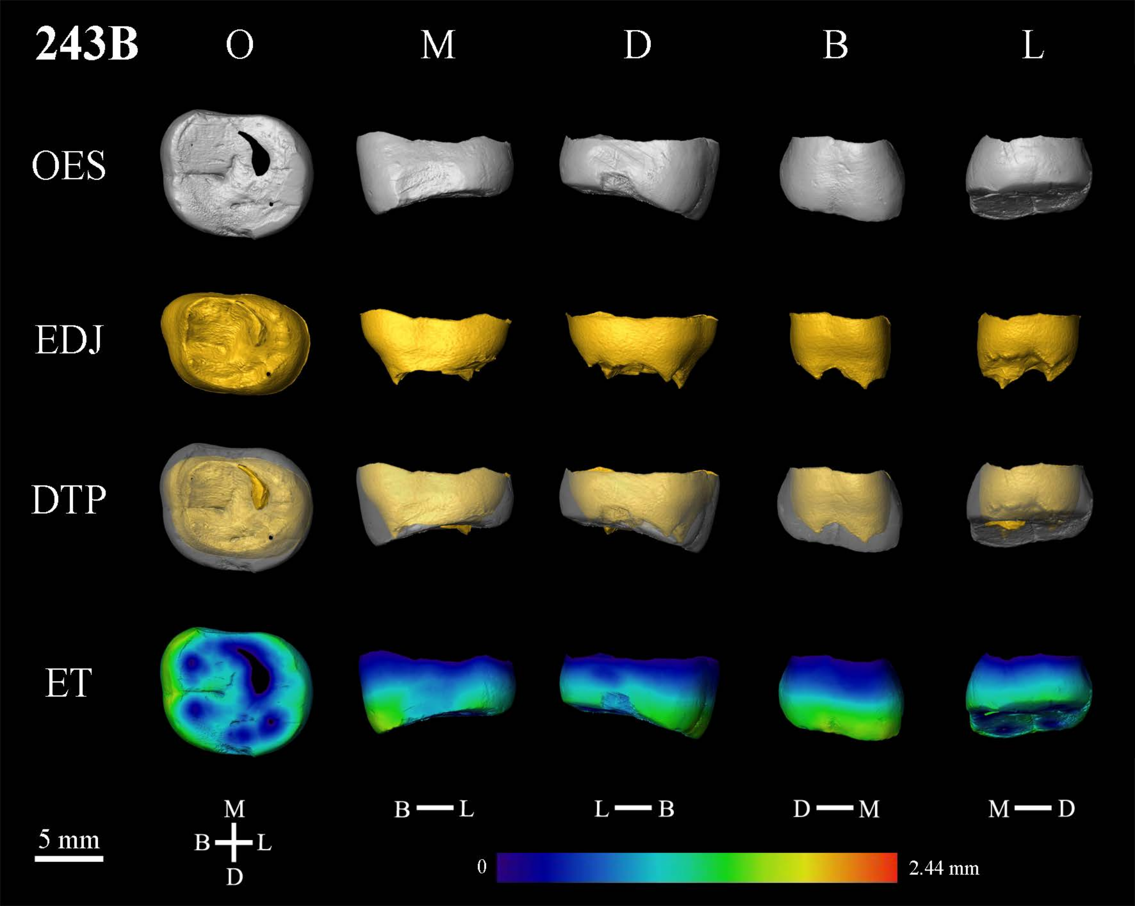
243B
O
M
D
B
L
OES
EDJ
DTP
ET
M
B L
D
B — L
L — B
D — M
M — D
5 mm
0
2.44 mm

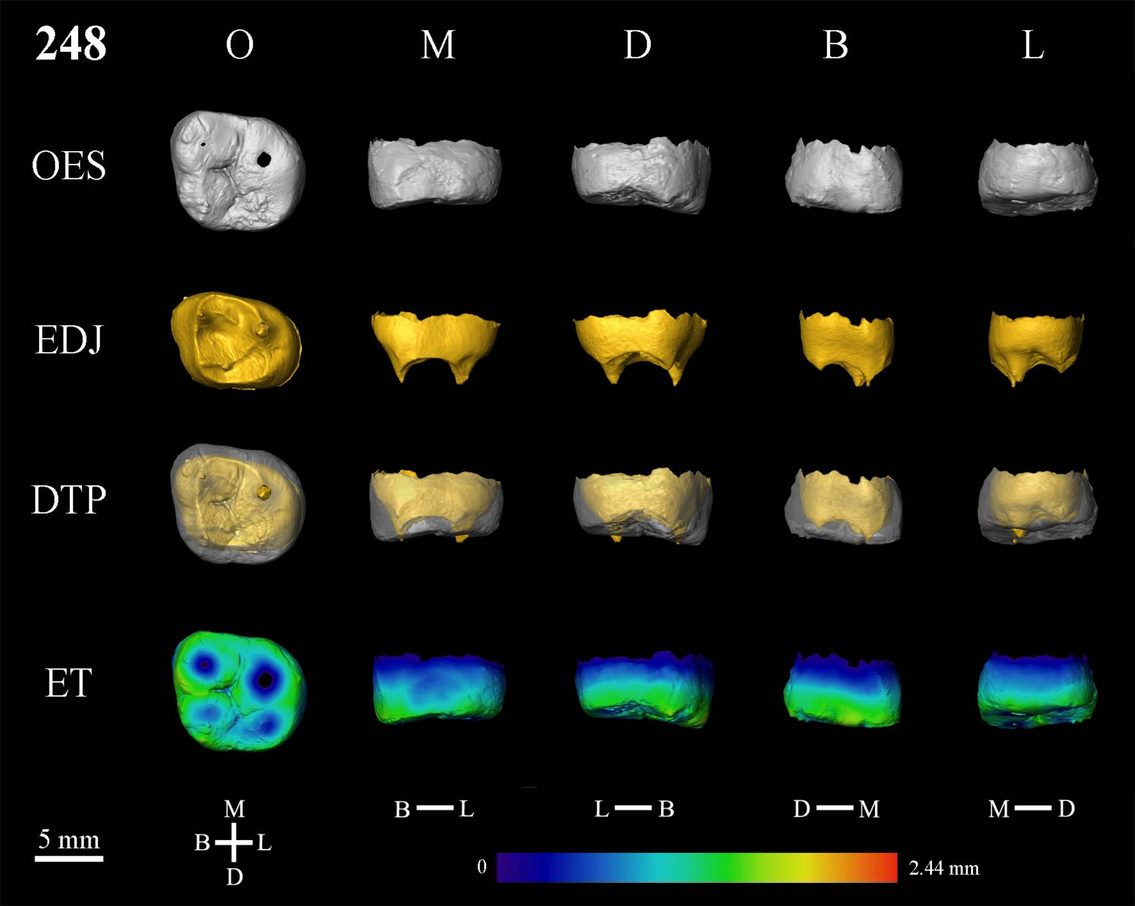
248
O
M
D
B
L
OES
EDJ
DTP
ET
M
B
L
D
B — L
L — B
D — M
M — D
5 mm
0
2.44 mm

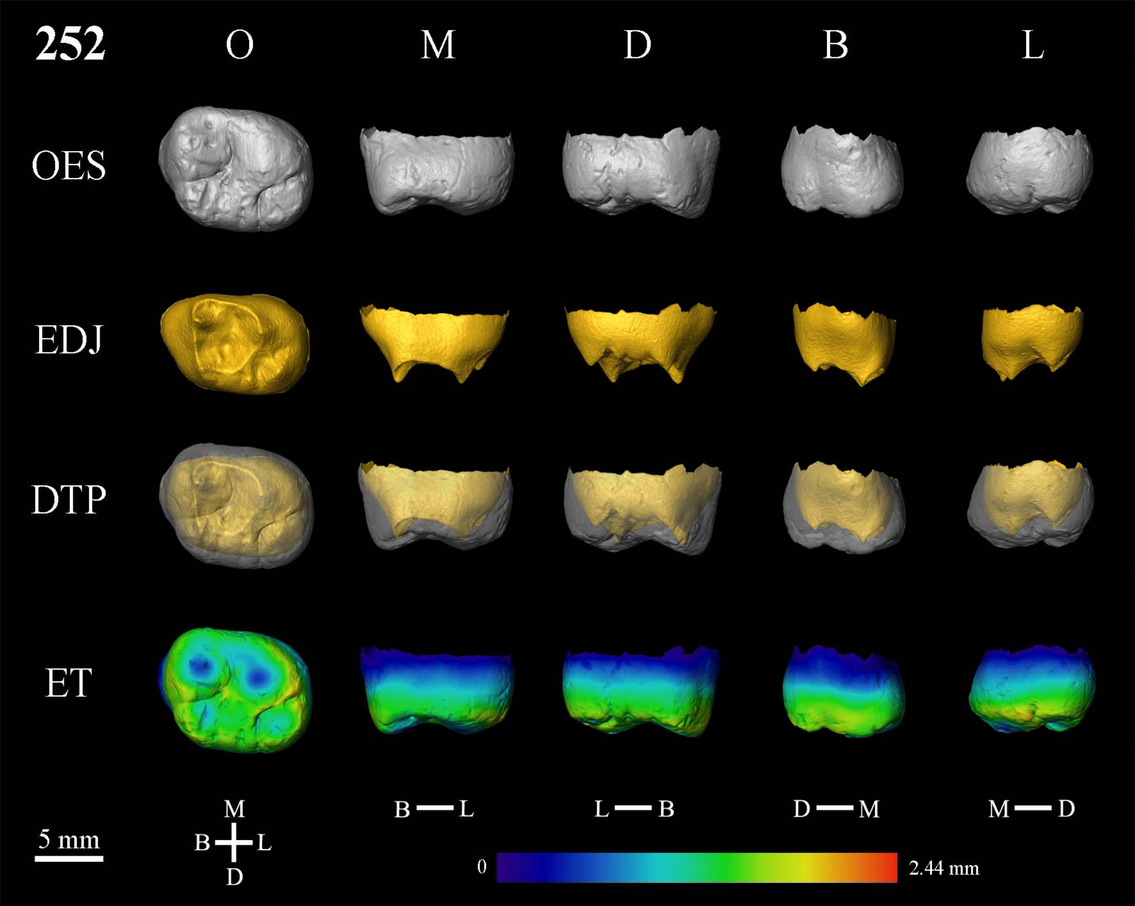
252
O
M
D
B
L
OES
EDJ
DTP
ET
M
B L
D
B L
L B
D M
M D
5 mm
0
2.44 mm

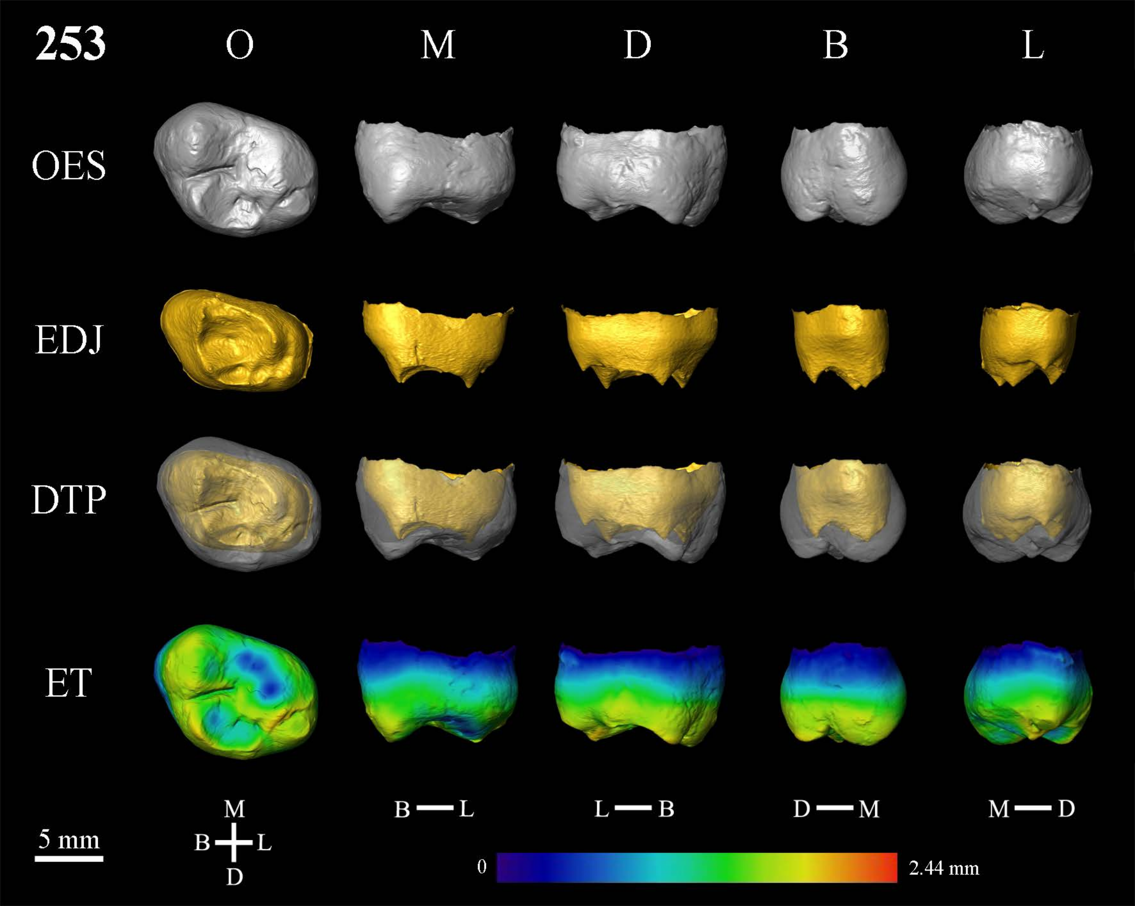
253
O
M
D
B
L
OES
EDJ
DTP
ET
M
B
L
D
B — L
L — B
D — M
M — D
5 mm
0
2.44 mm

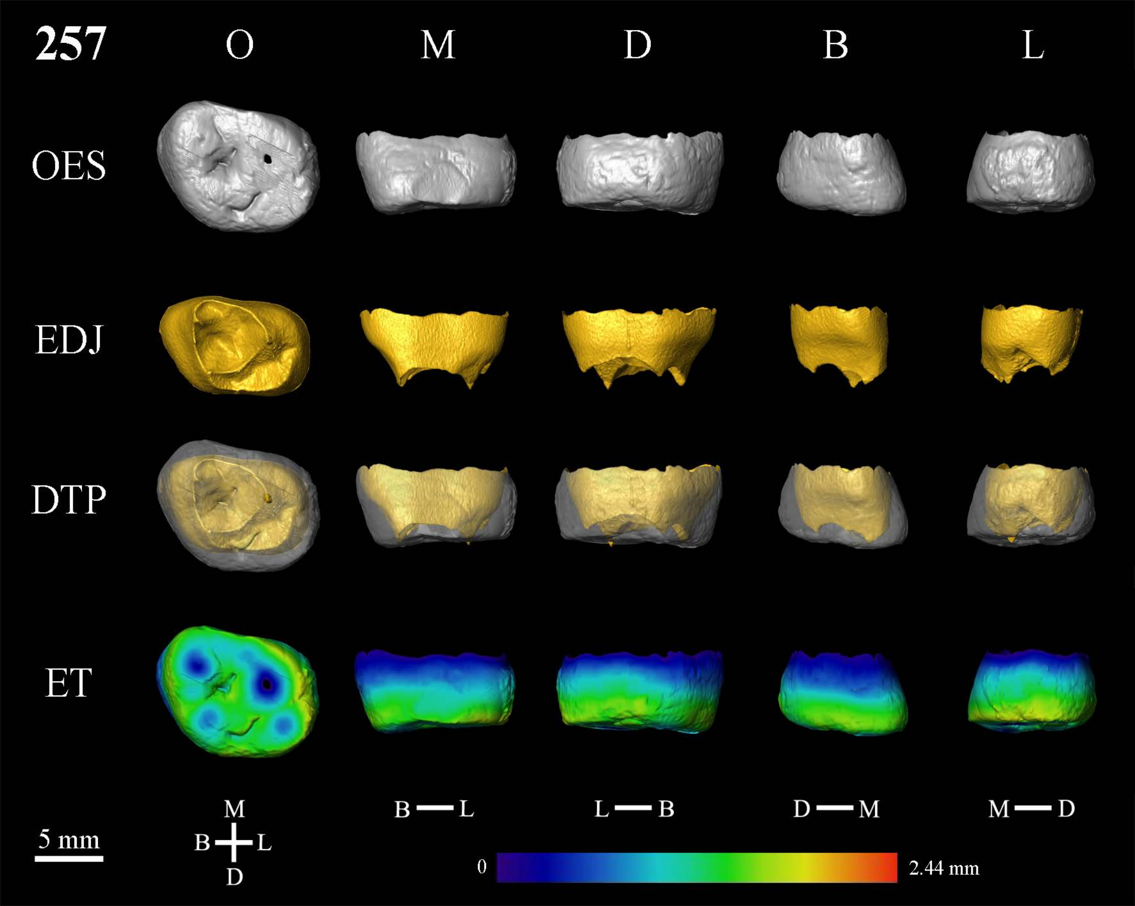

257
O
M
D
B
L
OES
EDJ
DTP
ET
M
B L
D
B — L
L — B
D — M
M — D
5 mm
0
2.44 mm

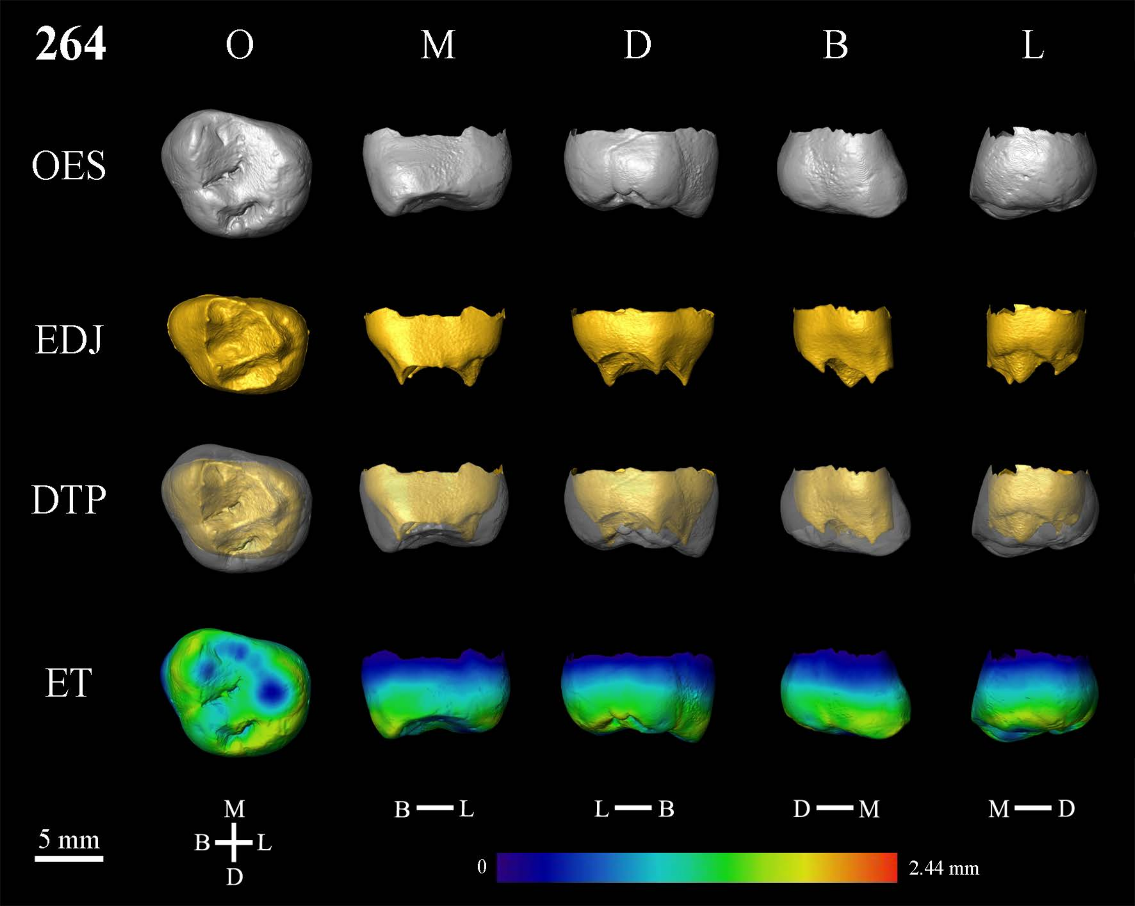
264
O
M
D
B
L
OES
EDJ
DTP
ET
M
B
L
D
B
L
L
B
D
M
M
D
5 mm
0
2.44 mm

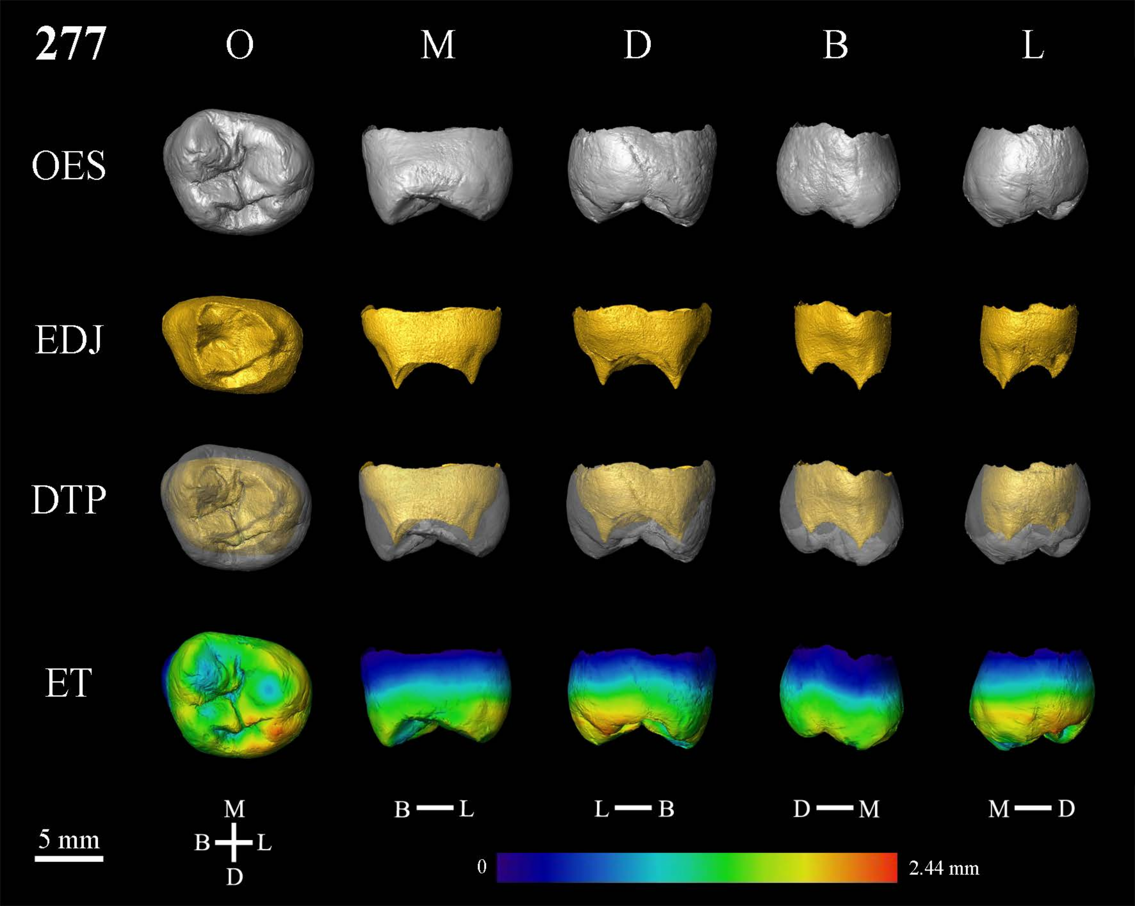

277
O
M
D
B
L
OES
EDJ
DTP
ET
M
B L
D
B L
L B
D M
M D
5 mm
0
2.44 mm

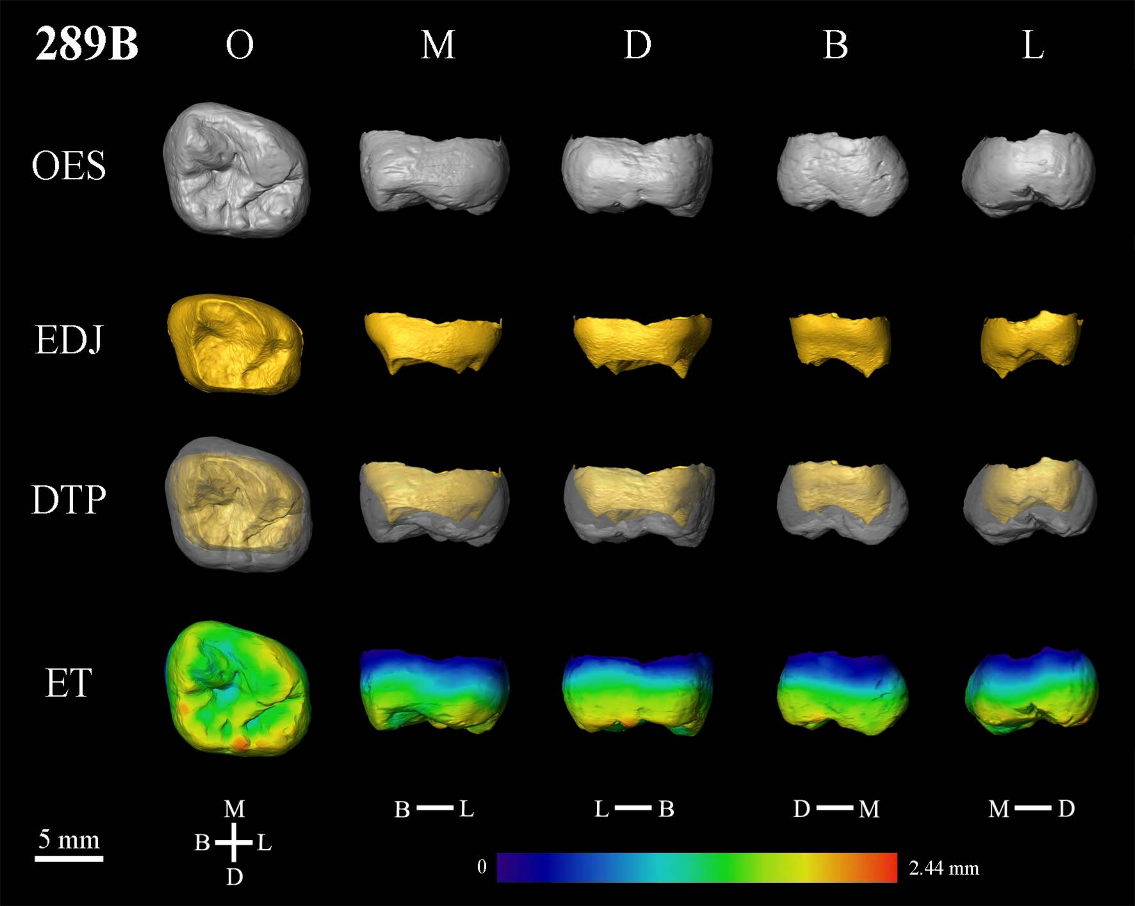
289B
O
M
D
B
L
OES
EDJ
DTP
ET
M
B L
D
B L
L B
D M
M D
5 mm
0
2.44 mm

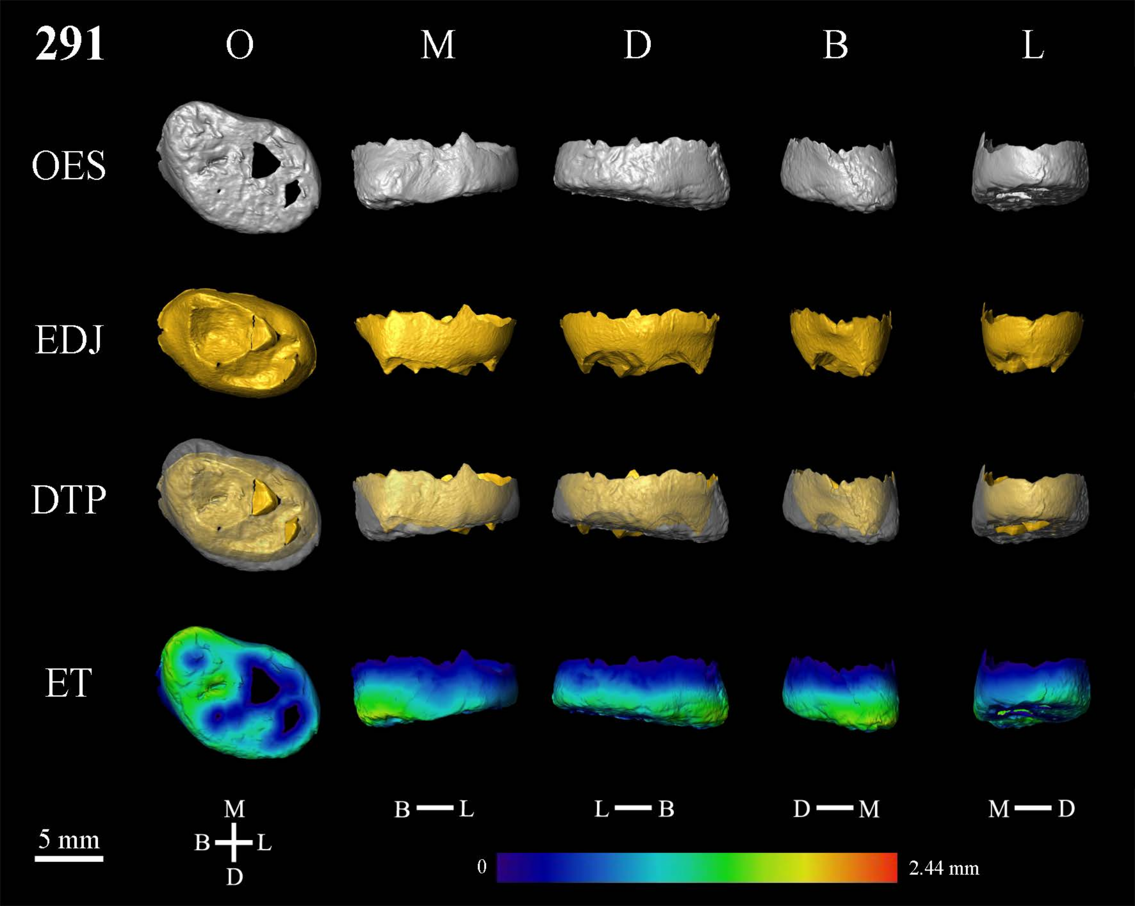
291
O
M
D
B
L
OES
EDJ
DTP
ET
M
B
L
D
B L
L B
D M
M D
5 mm
0
2.44 mm

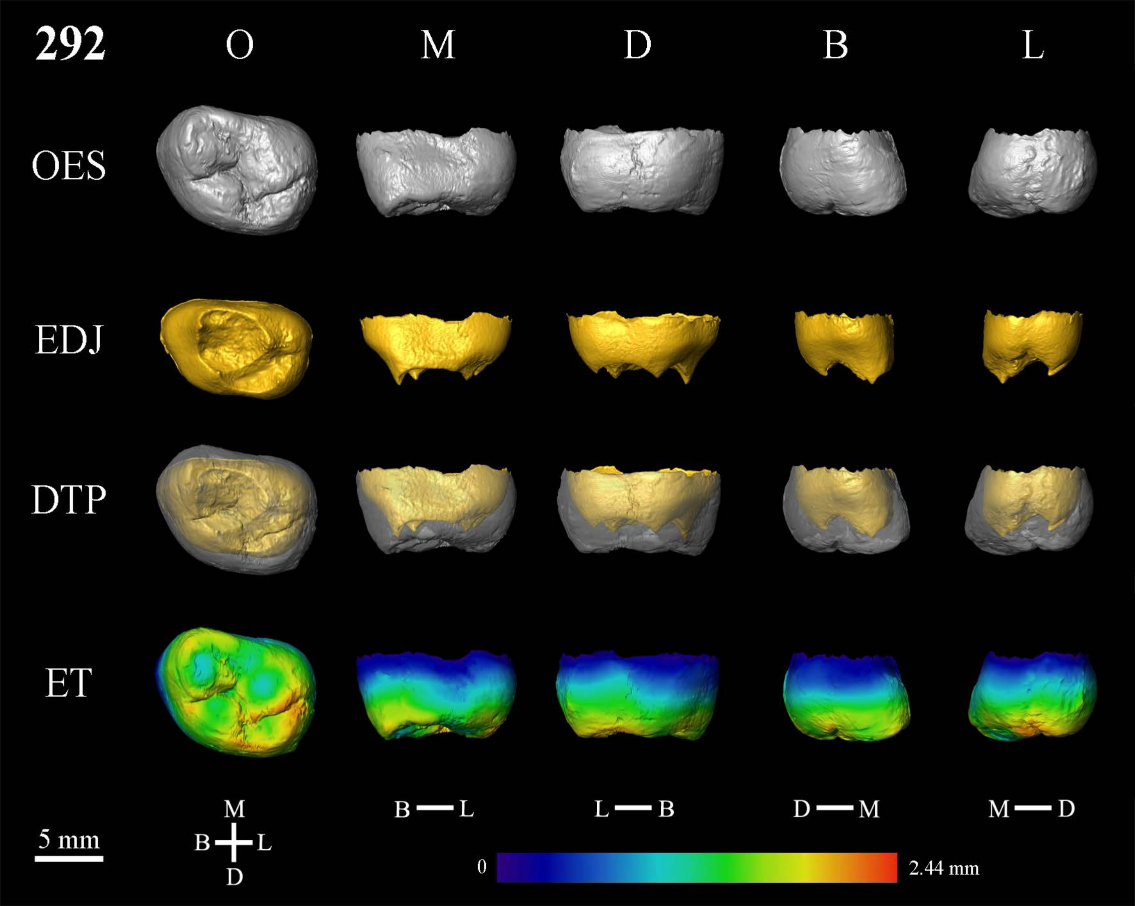
292
O
M
D
B
L
OES
EDJ
DTP
ET
M
B
L
D
B — L
L — B
D — M
M — D
5 mm
0
2.44 mm

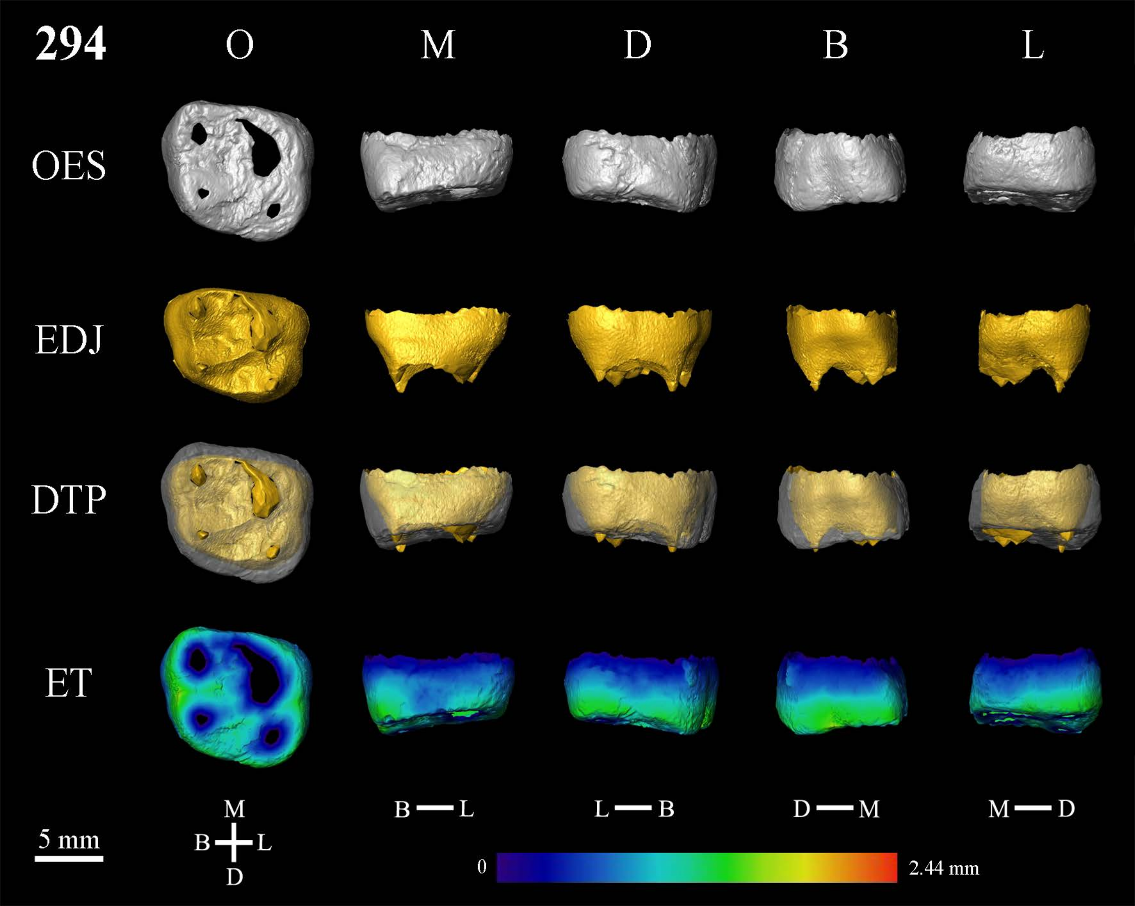
294
O
M
D
B
L
OES
EDJ
DTP
ET
M
B
L
D
B — L
L — B
D — M
M — D
5 mm
0
2.44 mm

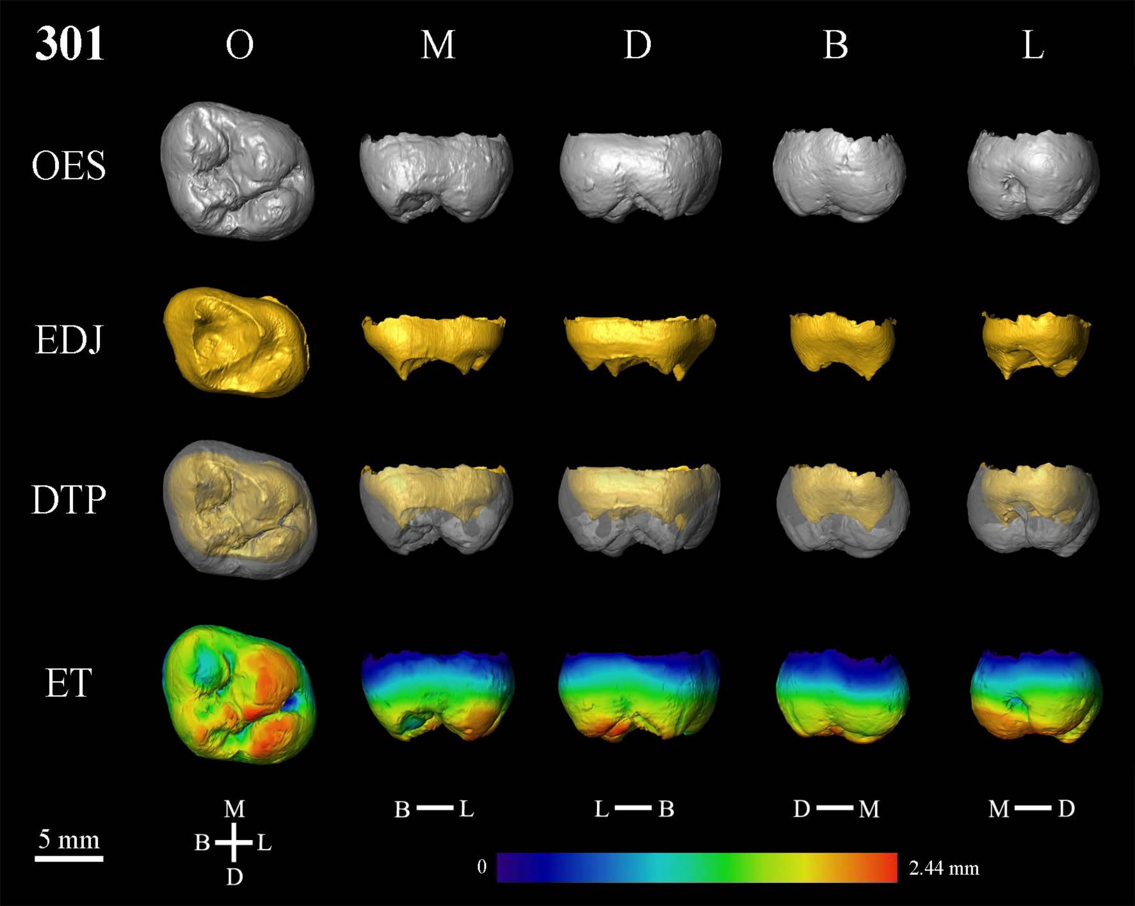
301
O
M
D
B
L
OES
EDJ
DTP
ET
M
B
L
D
B — L
L — B
D — M
M — D
5 mm
0
2.44 mm

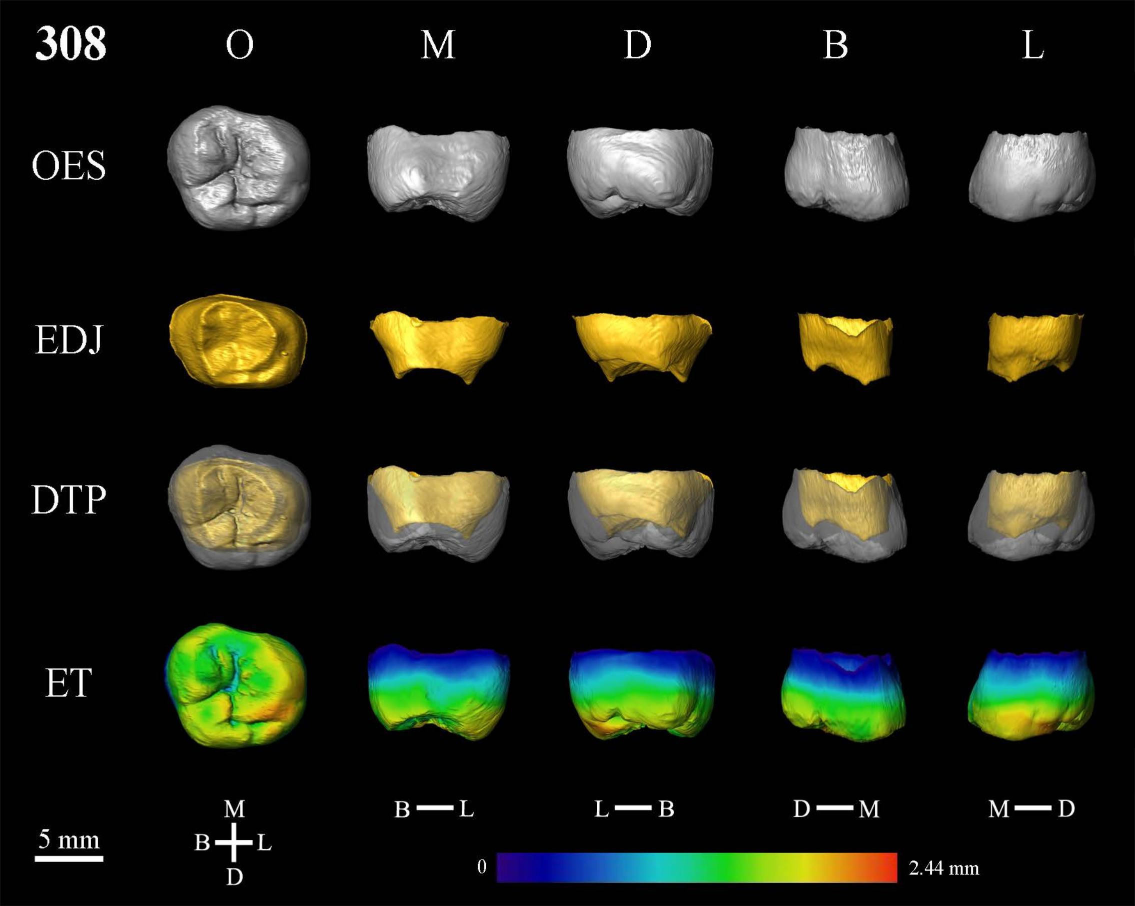
308
O
M
D
B
L
OES
EDJ
DTP
ET
M
B
L
D
B L
L B
D M
M D
5 mm
0
2.44 mm
